# *HCFC1* variants in the proteolysis domain are associated with X‐linked idiopathic partial epilepsy: Exploring the underlying mechanism

**DOI:** 10.1002/ctm2.1289

**Published:** 2023-06-01

**Authors:** Na He, Bao‐Zhu Guan, Jie Wang, Han‐Kui Liu, Yong Mao, Zhi‐Gang Liu, Fei Yin, Jing Peng, Bo Xiao, Bei‐sha Tang, Dong Zhou, Guang Huang, Qi‐Lin Dai, Ying Zeng, Hong Han, Qiong‐Xiang Zhai, Bin Li, Bin Tang, Wen‐Bin Li, Wang Song, Liu Liu, Yi‐Wu Shi, Bing‐Mei Li, Tao Su, Peng Zhou, Xiao‐Rong Liu, Li‐Wu Guo, Yong‐Hong Yi, Wei‐Ping Liao

**Affiliations:** ^1^ Department of Neurology, Institute of Neuroscience, Key Laboratory of Neurogenetics and Channelopathies of Guangdong Province and the Ministry of Education of China The Second Affiliated Hospital, Guangzhou Medical University Guangzhou China; ^2^ BGI‐Genomics, BGI‐Shenzhen Shenzhen China; ^3^ Frasergen Bioinformatics Co., Ltd Wuhan China; ^4^ Department of Pediatrics, Affiliated Foshan Maternity and Child Healthcare Hospital Southern Medical University Foshan China; ^5^ Department of Pediatrics, Xiangya Hospital Central South University Changsha China; ^6^ Department of Neurology, Xiangya Hospital Central South University Changsha China; ^7^ Department of Neurology, West China Hospital Sichuan University Chengdu China; ^8^ Department of Pediatrics The First Affiliated Hospital of Shantou University Medical College Shantou China; ^9^ Department of Neurology, The First Affiliated Hospital Sun Yat‐sen University Guangzhou China; ^10^ Department of Pediatrics Children's Hospital of Shanxi Taiyuan China; ^11^ Department of Pediatrics Guangdong General Hospital Guangdong Academy of Medical Sciences Guangzhou China; ^12^ Division of molecular testing Bio Diagnostic laboratories Brooklyn New York USA

**Keywords:** cobalamin metabolism disorders, *HCFC1* variant, molecular sub‐regional effect, partial epilepsy, proteolysis dysfunction

## Abstract

**Background:**

*HCFC1* encodes transcriptional co‐regulator HCF‐1, which undergoes an unusual proteolytic maturation at a centrally located proteolysis domain. *HCFC1* variants were associated with X‐linked cobalamin metabolism disorders and mental retardation‐3. This study aimed to explore the role of *HCFC1* variants in common epilepsy and the mechanism underlying phenotype heterogeneity.

**Methods:**

Whole‐exome sequencing was performed in a cohort of 313 patients with idiopathic partial (focal) epilepsy. Functional studies determined the effects of the variants on the proteolytic maturation of HCF‐1, cell proliferation and *MMACHC* expression. The role of *HCFC1* variants in partial epilepsy was validated in another cohort from multiple centers.

**Results:**

We identified seven hemizygous *HCFC1* variants in 11 cases and confirmed the finding in the validation cohort with additional 13 cases and six more hemizygous variants. All patients showed partial epilepsies with favorable outcome. None of them had cobalamin disorders. Functional studies demonstrated that the variants in the proteolysis domain impaired the maturation by disrupting the cleavage process with loss of inhibition of cell growth but did not affect *MMACHC* expression that was associated with cobalamin disorder. The degree of functional impairment was correlated with the severity of phenotype. Further analysis demonstrated that variants within the proteolysis domain were associated with common and mild partial epilepsy, whereas those in the kelch domain were associated with cobalamin disorder featured by severe and even fatal epileptic encephalopathy, and those in the basic and acidic domains were associated with mainly intellectual disability.

**Conclusion:**

*HCFC1* is potentially a candidate gene for common partial epilepsy with distinct underlying mechanism of proteolysis dysfunction. The HCF‐1 domains played distinct functional roles and were associated with different clinical phenotypes, suggesting a sub‐molecular effect. The distinct difference between cobalamin disorders and idiopathic partial epilepsy in phenotype and pathogenic mechanism, implied a clinical significance in early diagnosis and management.

## Graphical abstract

1. *HCFC1* is a candidate gene for common partial epilepsy.

2. Variants in the proteolysis domain disrupted the proteolytic maturation of HCF‐1 and the inhibition of cell proliferation.

3. The HCF‐1 domains played distinct functional roles and were associated with different clinical phenotypes.

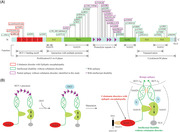

## 1 INTRODUCTION

The host cell factor C1 gene (*HCFC1*, MIM *300019) is located at Xq28 and encodes HCF‐1, which is ubiquitously expressed across the whole lifespan with predominant expression in the embryonic brain and associated with psychomotor development.^1^, ^2^ Mature HCF‐1 is a heterodimeric complex formed by its own N‐ and C‐terminal fragments after proteolysis.^3^ As a transcriptional coregulator, HCF‐1 plays critical roles in multiple biological processes, such as cell proliferation, migration and cell death.^4^ In embryonic murine neural cells, over‐expression of HCF‐1 reduces hippocampal neuronal arborization and increases neurotoxicity,^2^ while knockdown of HCF‐1 results in expansion of neural progenitor cells and promotes axonal growth of post‐mitotic neurons.^5^ In mice, knockout *Hcfc1* allele in the epiblast of male embryos leads to embryonic lethality.^6^

In humans, *HCFC1* variants have been associated with X‐linked recessive cobalamin metabolism disorder that presented severe epileptic encephalopathy, intellectual disability, failure to thrive and even early death (designated CblX, MIM #309541)^7^, ^8^ and also have been reported in intellectual disability with/without epilepsy but without metabolic disorders.^2^, ^9^, ^10^ It is suspected that *HCFC1* variants were associated with common epilepsy and the relationships between cobalamin metabolism disorders, intellectual disability and epilepsy remain elusive. It is noted that mature HCF‐1 complex possess a specific structure with distinct function of each domain. It is unknown whether the phenotype variations are associated with the functional domains.

In this study, trio‐based whole exome sequencing (WES) was performed in a cohort of patients with epilepsies of unknown causes (idiopathic). Seven hemizygous *HCFC1* variants were identified in 11 unrelated cases with idiopathic partial (focal) epilepsies without cobalamin disorder. Functional studies were performed to determine the effects of the variants on the proteolytic maturation of HCF‐1, cell proliferation and expression of metabolism of cobalamin associated C gene (*MMACHC*, MIM *609831). We confirmed the role of *HCFC1* variants in partial epilepsy in another cohort from multiple centers. The molecular sub‐regional effects of *HCFC1* variants were analysed.

## 2 MATERIALS AND METHODS

### 2.1 Patients

A total of 463 unrelated cases of epilepsy without acquired causes were consecutively enrolled from the Epilepsy Center of the Second Affiliated Hospital of Guangzhou Medical University from 2012 to 2019. The cohort consisted of 313 cases with idiopathic partial epilepsies and 150 cases with idiopathic generalized epilepsies. Brain MRI (magnetic resonance imaging) was performed to exclude symptomatic epilepsy. Video‐EEG (electroencephalogram) was performed and reviewed by two qualified electroencephalographers. Epileptic seizures and epilepsy were diagnosed and classified according to the criteria of the Commission on Classification and Terminology of the International League Against Epilepsy (1989, 2001, 2010 and 2017). As normal controls (in‐house), 296 healthy Chinese volunteers were recruited as in our previous report.^11^, ^12^

To verify the role of *HCFC1* variants in epilepsy, additional 80 cases with idiopathic partial epilepsy were collected from other seven hospitals in China.

This study received approval from the ethics committee of the Second Affiliated Hospital of Guangzhou Medical University, and adhered to the guidelines of the International Committee of Medical Journal Editors with regard to patient consent for research or participation. All participants and their parents provided written informed consents.

### 2.2 Whole exome sequencing

Genomic DNAs from blood samples of the patients and their parents (trios), and other familial members if available, were collected and used for segregation analysis. WES was performed on the Illumina HiSeq 2000 system by BGI‐Shenzhen (Shenzhen, China). The sequencing data were generated by massive parallel sequencing with >125 times average depth and >98% coverage of the capture regions. Sequence alignment and variant calling were performed according to standard procedures as previously described.^12^, ^13^ Potential disease‐causing variants were screened and evaluated case‐by‐case under five models^12^: (1) epilepsy‐associated gene; (2) dominant/de novo; (3) autosomal recessive inheritance including homozygous and compound heterozygous variants; (4) X‐linked; and (5) co‐segregation analysis. Genes with recurrent de novo variants, biallelic variants, hemizygous variants or variants with segregations, were considered for further studies to define the gene‐disease association. *HCFC1* was selected due to recurrent hemizygous mutations in the trio‐based cohort of partial epilepsy. The candidate variants were validated by Sanger sequencing. All *HCFC1* variants were annotated based on the transcript NM_005334.2.

### 2.3 Expression construct and transfection

The coding sequences of human *HCFC1* (NM_005334.2) and amino‐terminal HA epitope tag were cloned into pcDNA3.1(+) expression vectors. Mutant constructs were generated using the ClonExpressII One Step Cloning Kit (Agilent Technologies) and transfections were conducted using Lipofectamine 2000 (Invitrogen). Primer sequences are available upon request. All variants were verified by direct sequencing.

### 2.4 HCF‐1 proteolytic cleavage assay

To study HCF‐1 proteolysis processing, full‐length wild‐type and mutant HCF‐1 were expressed transiently in HEK293T cells and epitope‐tagged amino‐terminal polypeptides were subsequently detected by immunoblotting. Briefly, 48 h after transfection, approximately 80 µg of protein extracts were separated on 7.5% sodium dodecyl sulfate polyacrylamide gel electrophoresis (SDS‐PAGE) and transferred to PVDF membranes. Blots were blocked with 5% skimmed milk and then probed with 1:1000 anti‐HA tag antibody (Abcam, ab9110) at 4°C overnight. After washes, the blots were incubated with the appropriate secondary antibody (Proteintech, SA00001‐2) conjugated to horseradish peroxidase for 2 h. The immunodetection was performed using the chemiluminescent enhanced chemiluminescence reagents (Bio‐Rad).

### 2.5 Liquid chromatography mass spectrometry identification

To identify the proteolytic products, the immunoprecipitation and mass spectrometry analysis were conducted. Briefly, 48 h after transfection, HEK293T cells were lysed on ice in IP Lysis Buffer. After centrifugation, 1000 μg of supernatant was incubated with 10 μg anti‐HA (Abcam, ab9110) and 1 µg normal mouse anti‐IgG antibody (CST, 5415S) overnight at 4°C, and then incubated with 20 μL of protein G/A magnetic beads for 4 h at 4°C. After washes, the proteins were resuspended and then separated on a 7.5% SDS‐PAGE stained with silver stain (Beyotime). The stained protein bands were excised and trypsinized for analyses with liquid chromatography (LC) in combination with electrospray ionization‐tandem mass spectrometry (MS/MS) on a Triple time of flight (TOF) 5600+LC/MS system (AB SCIEX). The raw MS/MS data were analysed using ProteinPilot (version 4.5).

### 2.6 Quantitative real time polymerase chain reaction (qRT‐PCR)

To study the effect of *HCFC1* variants on cobalamin metabolism, we examined *MMACHC* expression. Total RNA was isolated from cultured HEK293T cells with HiPure Total RNA Kits (Magen), and cDNA was generated using Toyobo's ReverTra Ace qPCR RT Kit (Toyobo). Quantitative PCR analysis (200 ng) was performed using Thunderbird SYBR qPCR Mix (Toyobo) in a LightCycler 480 (Roche) system. The relative gene expression was calculated using the 2‐∆∆C_T_ method with *GAPDH* as an internal control. Primers for *GAPDH*, *HCFC1* and *MMACHC* are available upon request.

### 2.7 Immunofluorescence

To observe the sub‐localization of wild‐type and mutant HCF‐1, we employed immunofluorescent staining and confocal laser scanning microscopy. Forty‐eight hours after transfection, the fixed and blocked cells were incubated overnight at 4°C with the 1:500 primary antibodies (Abcam, ab9110) and then incubated with appropriate Alexa‐488‐conjugated secondary antibodies (Abcam, ab150077) for 2 h. After rinsing, cells were counterstained with DAPI. Fluorescence was visualized by confocal microscopy on a Leica TCS, SP8 microscope.

### 2.8 Cell proliferation assay

To examine the effect of *HCFC1* variants on cell proliferation, the growth rate of HEK293T cells was determined using the Cell Counting Kit‐8 (CCK‐8; Beyotime). Briefly, HEK293T cells or transfected HKE293T cells were seeded in 96‐well plates at a density of 5 × 10^3^ cells/well and cultured for 48 h at 37°C, and treated with 10 μl/well CCK‐8 solution (5 mg/ml) for 4 h. The absorbance was measured at 450 nm using a microplate reader (Tecan). Six parallel wells were set in each group, and the mean value was obtained.

### 2.9 Statistical analysis

The Student's *t*‐test was used to compare two independent samples, and one‐way ANOVA analysis was applied to compare multiple samples. Two‐tailed Fisher's exact test was used to compare allele frequencies between groups. Statistical analyses were performed with R statistical software (v4.0.3) and SPSS statistics 26.0. The *p* value < .05 was considered statistically significant.

## 3 RESULTS

### 3.1 Identification of *HCFC1* variants

**FIGURE 1:**
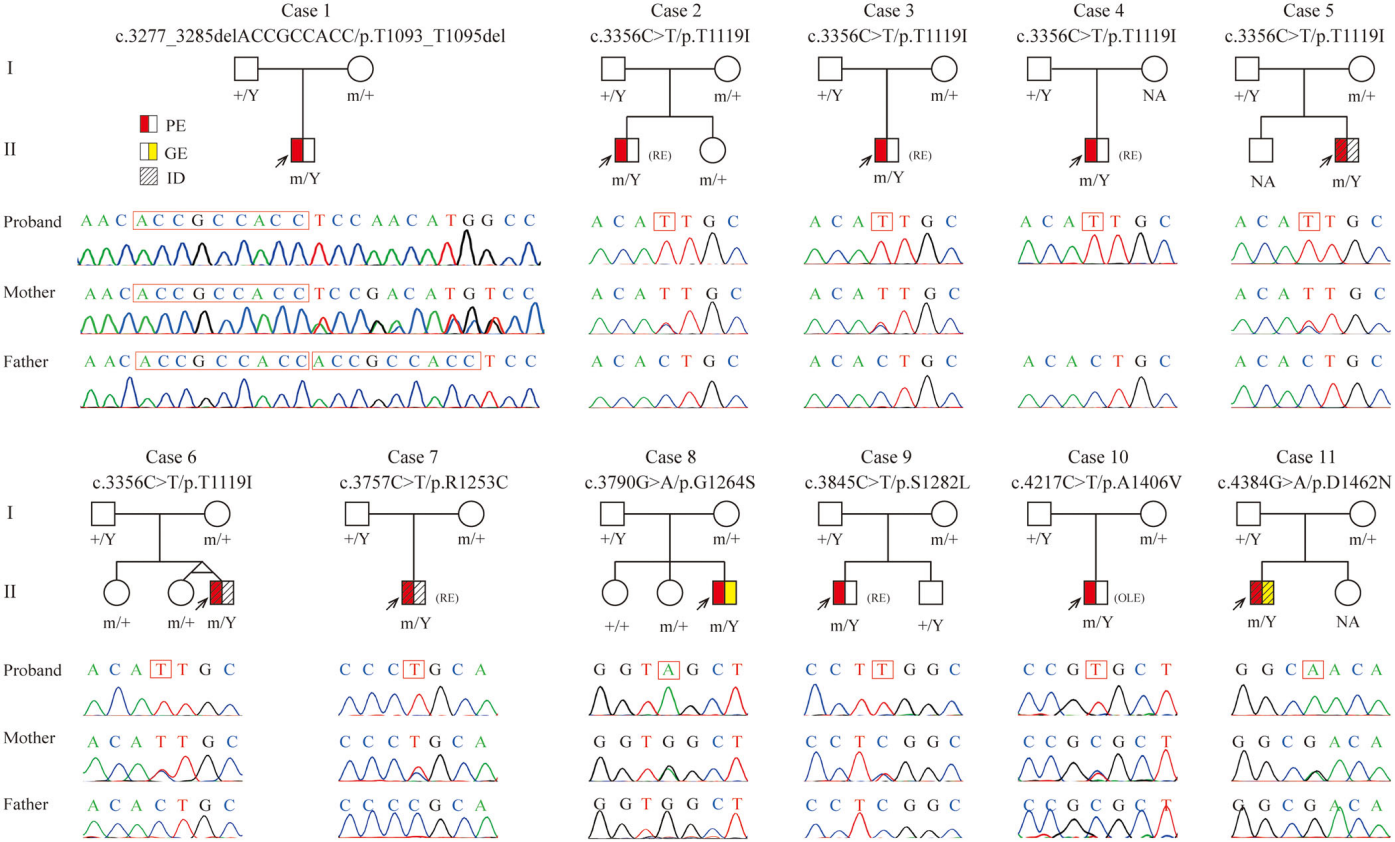
**Genetic data of cases with *HCFC1* variants**. Pedigrees and DNA sequencing chromatogram of the eleven cases with *HCFC1* variants and their corresponding phenotypes. Individuals with mutation were indicated by m/+ or m/Y, and those negative for mutation were indicated by +/Y. GE, generalized epilepsy; ID, intellectual disability; NA, not available; OLE, occipital lobe epilepsy; PE, partial epilepsy; RE, Rolandic epilepsy.

Seven hemizygous *HCFC1* variants were identified in 11 unrelated patients in our trio‐based cohort of 313 cases with idiopathic partial epilepsies. The hemizygous variants included c.3277_3285delACCGCCACC/p.Thr1093_Thr1095del, c.3356C > T/p.Thr1119Ile, c.3757C > T/p.Arg1253Cys, c.3790G > A/p.Gly1264Ser, c.3845C > T/p.Ser1282Leu, c.4217C > T/p.Ala1406Val and c.4384G > A/p.Asp1462Asn. The p.Thr1119Ile was recurrently detected in five cases (Figure 1 and Table 1).

**TABLE 1:** Clinical and genetic features of the patients with *HCFC1* variants in our cohort.

														Metabolism in blood		
Case	Variants	Origin	Sex	Age, yr	Age onset	Seizure course	EEG	Brain MRI	AEDs	Seizure‐ free (yr)	Diagnosis	ID	DD	Folate^a^	VB12^b^	Hcy^c^
1	c.3277_3285delACCGCCACC p.T1093_T1095del	Maternal	M	28	24 yr	FBTCS, 5 times during 2 yr; CPS, once	Normal	(‐)	VPA	2.5	PE	None	None	NA	NA	NA
2	c.3356C > T p.T1119I	Maternal	M	13	4 yr	CPS, 7 times during 6 mo; GTCS, twice	Right central‐temporal, left central spikes or SW	(‐)	VPA, LTG	8	RE	None	None	33.2	1087	10.0
3	c.3356C > T p.T1119I	Maternal	M	16	3 yr	GTCS twice during 1 yr; CPS, occasionally	Right central‐temporal spikes or SW	(‐)	LEV	8	RE	None	None	NA	NA	NA
4	c.3356C > T p.T1119I	NA	M	14	2 yr	SPS, FBTCS, 4 times during 6 yr	Left central‐temporal SW	(‐)	VPA	5	RE	None	None	NA	NA	NA
5	c.3356C > T p.T1119I	Maternal	M	25	4 yr	FBTCS ∼10 times/mo for age 4–6 yr; CPS, 1−5 times/mo for age 10−22 yr	Right or left temporal spikes or SW	(‐)	VPA, LTG	2	PE	Mild	None	15.2	422	10.0
6	c.3356C > T p.T1119I	Maternal	M	7	4 yr	CPS, 1−2 times/mo for 2 yr	Bilateral central‐temporal and right occipital SW	(‐)	VPA, LTG	1.5	PE	Mild	None	25.0	791	5.8
7	c.3757C > T p.R1253C	Maternal	M	7	8 mo	FBTCS ∼5 times/day for 3 yr; CPS, monthly	Bilateral central‐ parietal‐temporal SW	(‐)	LEV, CNZ	1	RE	Mild	None	18.0	579.7	5.4
8	c.3790G > A p.G1264S	Maternal	M	8	8 mo	FBTCS mostly triggered by fever, 3−4 times/yr for 6 yr; tonic, neglected	Left or right central‐parietal SW	(‐)	VPA	1.5	PE + GE	None	None	23.6	838	6.1
9	c.3845C > T p.S1282L	Maternal	M	8	3 yr	FBTCS, 7 times during 2 yr; CPS, 3 times	Right central‐ parietal‐temporal and frontal SW	(‐)	VPA, LTG, OXC	2.5	RE	None	None	35.1	975	5.4
10	c.4217C > T p.A1406V	Maternal	M	12	9 yr	CPS 1−4 times/mo for 1 yr	Left frontal‐central‐ parietal‐temporal SW	(‐)	LTG	2	OLE	None	None	18.8	366	8.2
11	c.4384G > A p.D1462N	Maternal	M	18	3 yr	GTCS, 1−2 times/mo at 3–5 yr; myoclonus/tonic, 1−6 times/day at 3–6 yr; CPS, occasionally	Left temporal SW; 2.5‐3.0 Hz GSW	(‐)	VPA, LTG, LEV	2.5	PE + GE	Mild	None	23.6	614	3.9

Abbreviations: CPS, complex partial seizure; CNZ, clonazepam; DD, developmental disorder; EEG, electroencephalography; FBTCS, focal to bilateral tonic‐clonic seizures; GE, generalized epilepsy; GSW, generalized spike‐and‐slow waves; GTCS, generalized tonic‐clonic seizure; Hcy, homocysteine; ID, intellectual disability; LEV, levetiracetam; LTG, lamotrigine; M, male; mo, months; MRI, magnetic resonance imaging; NA, not available; OLE, occipital lobe epilepsy; OXC, oxcarbazepine; PE, partial epilepsy; RE, Rolandic epilepsy; SPS, simple partial seizure; SW, spike‐and‐slow waves; VB12, vitamin B12; VPA, valproate; yr, years; (‐), negative.

^a^ Normal range is 15.9‐71.1 nmol/L.

^b^ Normal range is 138–652 pmol/L.

^c^ Normal range is 0−15.0 umol/L.

All the 11 patients with *HCFC1* variants were boys. In contrast, the female carriers with heterozygous variants were asymptomatic (Figure 1). It is consistent with the X‐linked recessive inheritance pattern, as the previously reported cases with *HCFC1* variants.^2^, ^5^, ^7^

No hemizygous or homozygous *HCFC1* variants were identified in the 150 patients with idiopathic generalized epilepsies.

The c.3790G > A/p.Gly1264Ser and c.4384G > A/p.Asp1462Asn were not present in any public databases, including the 1000 Genomes Project, Exome Aggregation Consortium, and gnomAD. The other five variants were observed with a very low allele frequency in the control populations of gnomAD as hemizygotes (Table 2). Homozygotes of these variants were not detected in general populations. All the variants were absent from our in‐house 296 normal controls. We compared the frequencies of these hemizygous variant alleles in the present cohort with that in control populations of gnomAD (Table 2). In this cohort, 11 hemizygous mutant alleles in a total of 438 alleles (.025114, 188 male and 125 female cases) were detected, which was significantly higher than that in the controls of the East Asian population (.002119, 14/6606, *p* = 9.6 × 10^−8^) and that in the controls of all populations in gnomAD (.000226, 18/76492, *p* = 2.2 × 10^−16^).

**TABLE 2:** Analysis of the aggregate frequency of *HCFC1* variants identified in this study.

Variants	Allele count/number in hemizygotes in this study	Allele count/number in hemizygotes of gnomAD‐all populations	Allele count/number in hemizygotes in controls of gnomAD‐all populations	Allele count/number in hemizygotes of gnomAD‐ East Asian populations	Allele count/number in hemizygotes in controls of gnomAD‐ East Asian populations
c.3277_3285delACCGCCACC/ p.T1093_T1095del	1/438 (.002283)	4/199736 (.00002)	1/86051 (.000012)	3/14363 (.000209)	0/7134 (0)
c.3356C > T/p.T1119I	5/438 (.011416)	23/199678 (.000115)	11/86019 (.000128)	23/14463 (.00159)	11/7157 (.001537)
c.3757C > T/p.R1253C	1/438 (.002283)	10/193599 (.000052)	4/84220 (.000047)	8/14371 (.000557)	3/7145 (.00042)
c.3790G > A/ p.G1264S	1/438 (.002283)	−/−	−/−	−/−	−/−
c.3845C > T/ p.S1282L	1/438 (.002283)	2/180301 (.000011)	1/79638 (.000013)	1/13540 (.000074)	0/6619 (0)
c.4217C > T/ p.A1406V	1/438 (.002283)	1/179615 (.000006)	1/79492 (.000013)	0/13506 (0)	0/6606 (0)
c.4384G > A/ p.D1462N	1/438 (.002283)	−/−	−/−	−/−	−/−
Total	11/438 (.025114)	40/179615 (.000223)	18/76492 (.000226)	35/13506 (.002591)	14/6606 (.002119)
*P* value		2.2 × 10^−16^	2.2 × 10^−16^	1.3 × 10^−7^	9.6 × 10^−8^
OR (95% CI)		115.5 (53.2–231.1)	113.3 (48.3–256.6)	9.9 (4.5–20.1)	12.1 (4.9–28.9)

*Note*: *p* Values and odds ratio were estimated with a two‐sided Fisher's exact test.

Abbreviations: CI, confidence interval; gnomAD, Genome Aggregation Database; OR, odds ratio.

None of the 11 cases had other pathogenic or likely pathogenic variants in genes known to be associated with seizures.^14^

The c.3277_3285delACCGCCACC/p.Thr1093_Thr1095del was potentially deleterious, yielding a 3‐amino‐acid deletion of the protein HCF‐1. The other six missense variants were predicted to be damaging by multiple predictors (Table S1). Three of the missense variants (p.Thr1119Ile, p.Ala1406Val and p.Asp1462Asn) changed their hydrogen bonds with surrounding residues (Figure S1B). We further studied the effects of *HCFC1* variants on the function of HCF‐1 experimentally.

### 3.2 Effect on proteolytic cleavage

**FIGURE 2:**
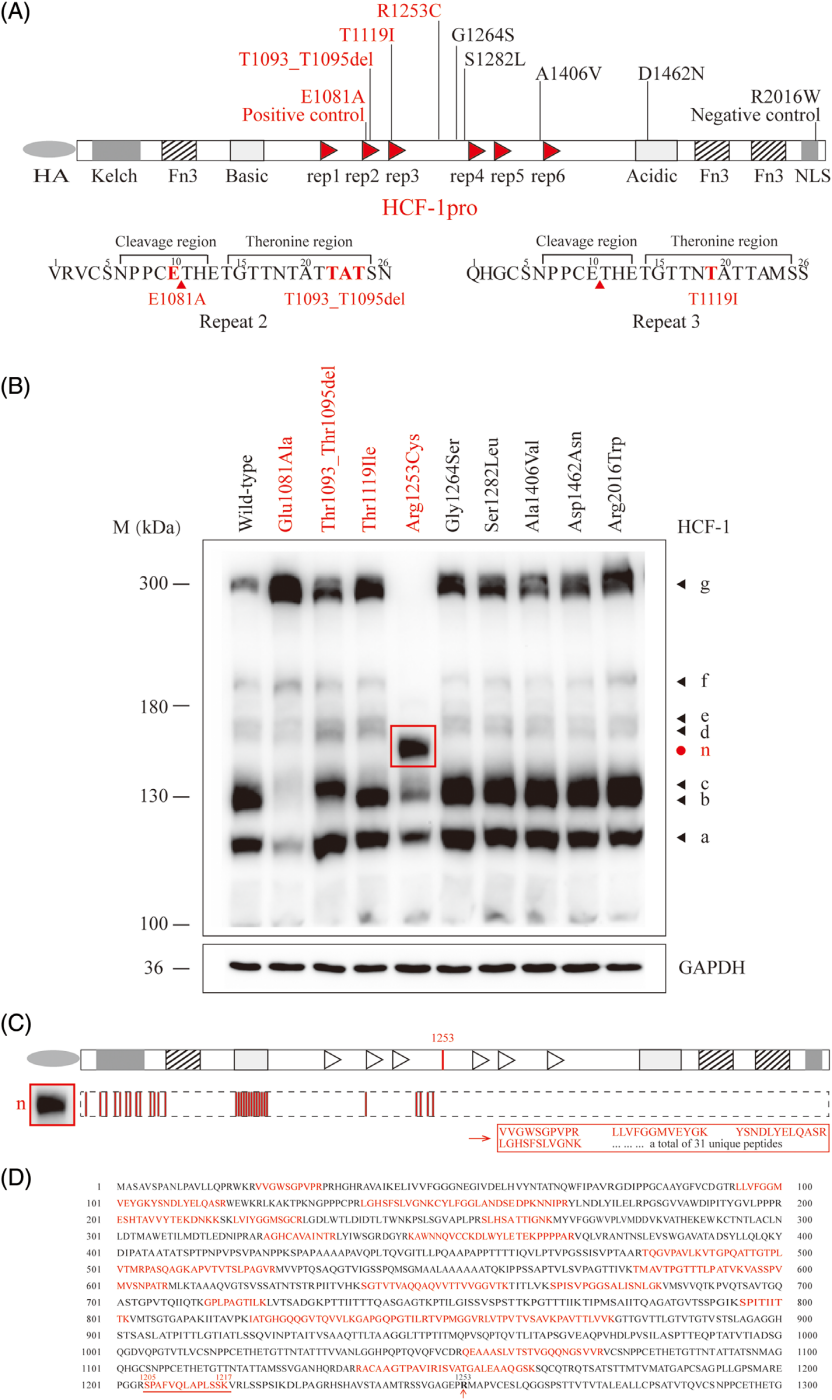
**Proteolytic cleavage analysis of *HCFC1* variants. (A)** Schematic illustration of the HA epitope‐tagged full‐length HCF‐1 protein and the locations of the *HCFC1* variants. HCF‐1 consists of the kelch domain, the basic domain, the proteolysis domain (HCF‐1_PRO_ repeats 1−6), the acidic domain, fibronectin 3 domains (Fn3), and the nuclear localization signal domain (NLS). Amino acid sequences of highly conserved HCF‐1_PRO_ repeats 2 and 3 include a cleavage and a threonine region. Red triangle (▲) indicates the cleavage sites. Locations of residues E1081, T1093_T1095 and T1119 in repeat 2 and 3 are indicated and highlighted in red. **(B)** Cleavage products of the wild‐type and mutant HCF‐1 proteins. The black arrowheads (◄) indicate HA epitope‐tagged products generated by the wild‐type HCF‐1 expression construct. The red dot (●) indicates a new major product of ∼140 kD. Molecular weight (MW) markers are given in kDa. GAPDH was used as a loading control. Variants with obvious abnormal cleavage products are highlighted in red. **(C)** Locations of the 31 unique HCF‐1 peptides in the protein band n identified by LC‐MS/MS. **(D)** Protein sequence and the 31 unique peptides. Red colored amino acids indicate protein coverage of 30.9% of the amino‐terminal fragment HCF‐1_1‐1253_ and 19.02% of the full‐length HCF‐1.

HCF‐1 precursor consists of a kelch domain, a basic domain, a proteolysis domain, an acidic domain, three fibronectin 3 domains (Fn3) and a nuclear localization signal (NLS) domain (Figure 2A). The proteolysis domain contains six centrally located and highly conserved 26‐amino‐acid repeats, named HCF‐1_PRO_ repeats 1−6. Each HCF‐1_PRO_ repeat contains a cleavage region (N6‐H12) and a threonine region (T14‐T24) that is a binding site of *O*‐linked β‐*N*‐acetylglucosamine transferase (OGT).^15^, ^16^ After binding, OGT will *O*‐GlcNAcylate the HCF‐1_N_ subunit and directly cleave the HCF‐1_PRO_ repeat at E10 cleavage site in a stochastic manner.^15^, ^16^

Six of the variants identified in this cohort, p.Thr1093_Thr1095del, p.Thr1119Ile, p.Arg1253Cys, p.Gly1264Ser, p.Ser1282Leu and p.Ala1406Val were located in the proteolysis region (Figure 2A). The variant p.Thr1093_Thr1095del yielded a deletion containing the essential binding threonine (T22) of the repeat 2, and potentially disrupting the binding with OGT. The variant p.Thr1119Ile substituted the essential binding threonine (T19) of the repeat 3, potentially leading to loss a binding point with OGT. The other four variants within the proteolysis region were located between the repeats. One of the variants, p.Asp1462Asn was located in the acidic domain.

To examine the effect of mutants on the cleavage, a positive control (c.3242A > C/p.Glu1081Ala) and a negative control (c.6046C > T/p.Arg2016Trp) were set. The mutation p.Glu1081Ala, which lies in the E10 cleavage site of the repeat 2, has previously been verified to block cleavage.^3^, ^15^, ^16^ The variant p.Arg2016Trp, which is located in the C‐terminal NLS domain and shown to disrupt the nuclear localization in the previous study,^5^ was theorized to have no effect on cleavage.

The wild‐type HCF‐1 protein generated at least six amino‐terminal HA epitope‐tagged polypeptides labeled a‐f (Figure 2B), similar to the previous studies.^3^, ^17^ The relative sizes of these six bands a to f potentially represent amino‐terminal cleavage products of repeats 1 to 6, respectively. The largest size of a ∼300 kD polypeptide (the band g) corresponds to the full‐length translation product HCF_300._ As shown in Figure 2B, the positive control p.Glu1081Ala resulted in the absence of the second and third shorter polypeptides (band b and c) compared to the wild‐type HCF‐1. Variants p.Thr1093_Thr1095del and p.Thr1191Ile, which were located in the canonical proteolytic sites in the threonine regions of the repeat 2 and repeat 3, respectively, resulted in the absence of the second (band b) and third (band c) smaller polypeptides, respectively. The variant p.Arg1253Cys was located between the repeats and near the center of the proteolysis domain and did not affect the formation of the first three shorter HCF‐1 polypeptides; but surprisingly, it resulted in a new major epitope‐tagged polypeptide of ∼140 kD (band n) with loss of the next three cleaved fragments (band d to f) and the largest protein HCF_300_ (band g). Three variants located between the repeats (p.Gly1264Ser, p.Ser1282Leu, and p.Ala1406Val), and p.Asp1462Asn in the acidic domain, did not disrupt the proteolytic processing compared to the wild‐type (Figure 2B and Figure S2).

The new proteolytic product of mutant p.Arg1253Cys (band n, indicated in the red box in Figure 2B) was excised and analysed by LC‐MS/MS. As shown in Figure 2C, the band n contained 31 unique HCF‐1 peptides (Table S2, Figure S3.1‐S3.31), which covered the amino acids before residue p.Arg1253 (indicated in red in Figure 2D). The last peptide sequence, SPAFVQLAPLSSK from amino acids 1205 to 1217, was 36 amino acids away from the residue p.Arg1253, suggesting the cleavage point was between the amino acids 1217 to 1253.

### 3.3 Effect on cell proliferation

**FIGURE 3:**
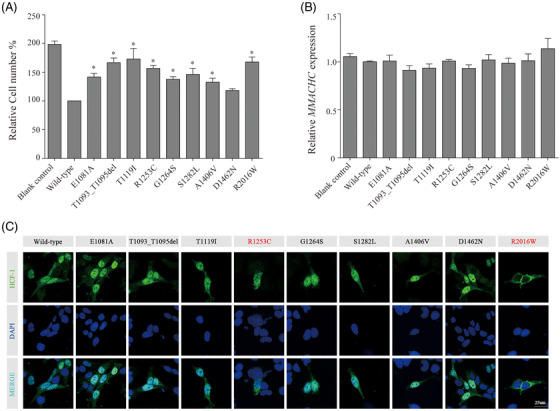
**Effect of *HCFC1* variants on cell proliferation, *MMACHC* expression and subcellular localization. (A)** *HCFC1* variants disrupted the inhibition of cell proliferation of HEK293T cells. **(B)** *HCFC1* variants did not affect the expression of *MMACHC*. The mRNA level of *MMACHC* was normalized to that of *GAPDH*. HEK293T cells were transfected with an empty expression vector (blank control) or with expression vectors encoding either wild‐type or mutant HCF‐1. **(C)** The subcellular localization of wild‐type and mutant HCF‐1. The HA epitope‐tagged wild‐type and mutant HCF‐1 proteins were expressed in HEK293T cells and detected through immunofluorescence using anti‐HA antibody (green). Cell nuclei were counterstained with DAPI (blue). Variants with failure in nuclear localization are highlighted in red; *n* = 18 cells for each condition from three different transfections. Data are representative of three independently replicate experiments and expressed as mean ± standard deviation (SD). *p*‐Values were calculated by Student's *t*‐test;*p  <  .05 is statistically significant from wild‐type.

HCF‐1 potentially inhibits cell proliferation via regulating cell‐cycle progression.^18^, ^19^ In this study, the growth rate of HEK293T cells was measured by CCK‐8 assay after 48 h of growth. The overexpressed wild‐type *HCFC1* resulted in a 50% decrease in growth compared with the blank control cultures (transfected with an empty expression vector), showing a distinct inhibition on cell proliferation (Figure 3A), consistent with the previous report.^5^ Comparing with the wild‐type *HCFC1*, all the nine variants caused increases in cell number with a range from 18% to 73%, indicating loss of growth suppression (Figure 3A). The increase was statistically significant in eight of nine variants. The p.Asp1462Asn mutant, which was located in the acidic domain, resulted in increases in growth of 18%, showing a modest, but not significant difference from wild‐type HCF‐1. The data suggest these variants may cause loss of function in inhibition of cell proliferation of various degrees.

### 3.4 Effect on *MMACHC* expression

*MMACHC*, one of downstream target genes regulated by *HCFC1*, encodes a cobalamin transport protein with enzymatic capabilities. Previous studies have revealed that *HCFC1* variants reduced *MMACHC* expression in fibroblasts derived from patients with cobalamin metabolism disorders.^4^, ^7^, ^20^ This study utilized HEK293T cells and over‐expressed wild‐type or mutant HCF‐1 and analysed the expression of *MMACHC* by qPCR as previously described.^5^ As shown in Figure 3B, the relative expression of *MMACHC* in the nine HEK293T cell lines with *HCFC1* variants were similar to that with wild‐type *HCFC1*, suggesting that these nine *HCFC1* variants did not affect the expression of *MMACHC*.

### 3.5 *HCFC1* variants on nuclear localization

As shown in Figure 3C, the wild‐type HCF‐1 was predominantly distributed in the nucleus. In contrast, the mutant p.Arg2016Trp, in which the mutation was located in the NSL and affected the nuclear localization, was enriched in the cytoplasm, as that in the previous study.^5^

The p.Arg1253Cys mutant also failed to localize to the nucleus and was detected predominantly in the cytoplasm, suggesting a loss of ability in nuclear localization. The other seven *HCFC1* variants were expressed mainly in the cell nuclei, akin to the wild type.

### 3.6 Clinical features of patients with *HCFC1* variants

**FIGURE 4:**
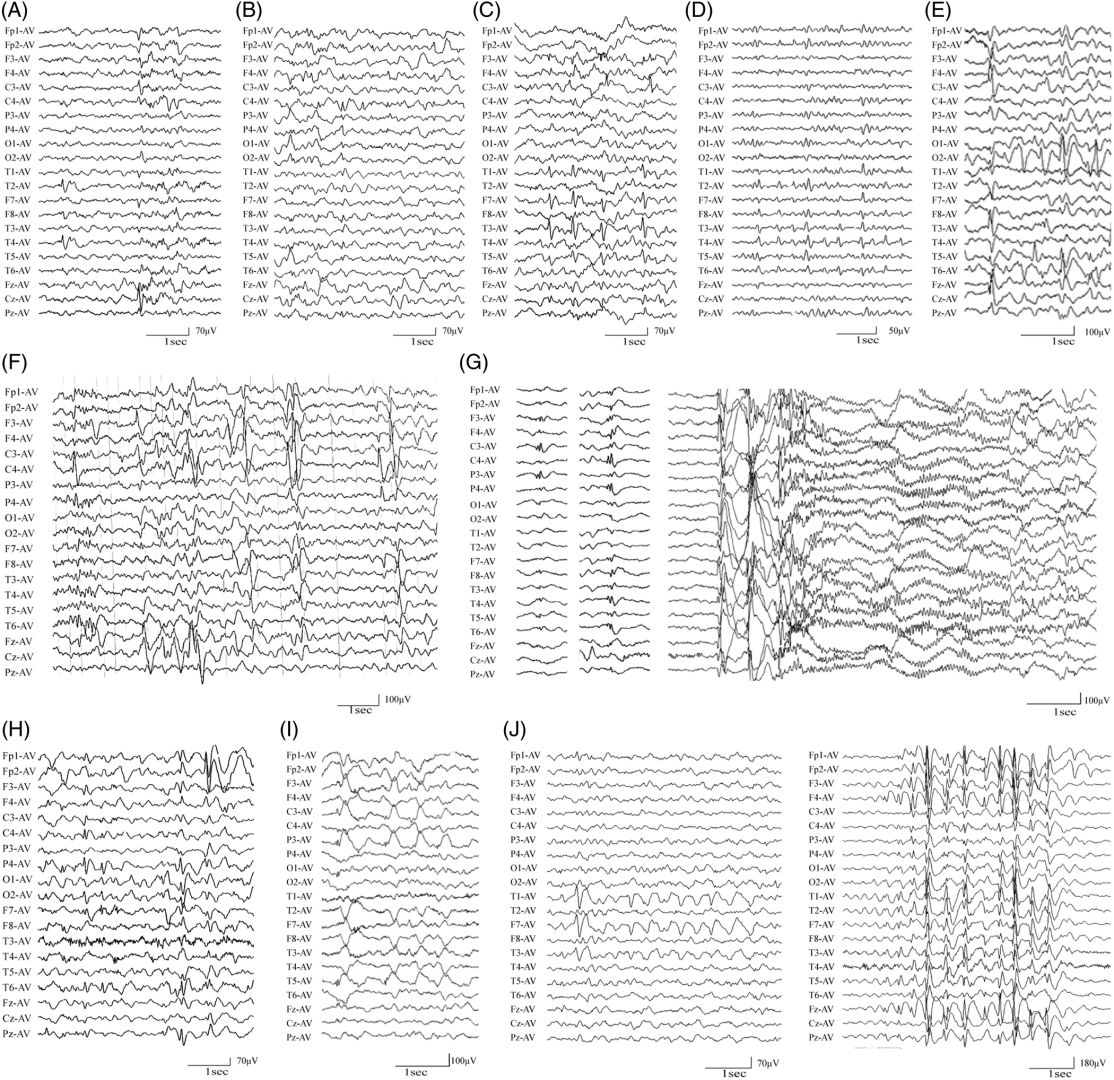
**Representative EEG recordings of the patients with *HCFC1* variants**. (A) Interictal EEG of case 2 showed independent spikes or spike‐and‐slow waves in the right mid‐temporal and central regions or in left central region. (B) Interictal EEG of case 3 showed spikes or spike‐and‐slow waves in the right central‐temporal regions. (C) Interictal EEG of case 4 showed repetitive spike‐and‐slow waves in the left central‐temporal regions. (D) Interictal EEG of case 5 showed spikes or spike‐and‐slow waves in the right or left temporal regions. (E) Interictal EEG of case 6 showed spike‐and‐slow waves in the bilateral central‐temporal regions and right occipital region. (F) Interictal EEG of case 7 showed spike‐and‐slow waves in the bilateral central‐parietal‐temporal regions (G) Interictal EEG of case 8 showed spikes or spike‐and‐slow waves in the left or right central‐parietal regions; and an ictal EEG showed a tonic seizure with generalized fast rhythms, lasting for 5 s. (H) Interictal EEG of case 9 showed spikes or spike‐and‐slow waves in the right central‐parietal‐temporal regions and the right frontal region. (I) Interictal EEG of case 10 showed spike‐and‐slow or slow waves in the left frontal‐central‐parietal‐temporal regions. (J) Interictal EEG of case 11 showed spike‐and‐slow or slow waves in the left temporal region and 2.5–3.0 Hz generalized spike‐and‐slow waves.

The clinical characteristics of the 11 cases with *HCFC1* variants are summarized in Table 1. The seizure onset age ranged from 8 months to 24 years old, with a median onset age of 3 years. All the patients with missense variants started seizures in infancy or childhood, while the patient with in‐frame deletion had late‐onset age of 24 years old. All the patients had focal seizures or focally originating tonic‐clonic seizures characterized by shifting or bilateral focal discharges (Figure 4). Among them, five cases were diagnosed as Rolandic epilepsy, one case as occipital lobe epilepsy, three cases as partial epilepsies without specific characteristic, and two cases of partial epilepsy with additional generalized seizures (case 8 and 11; Figure 1 and Table 1). Frequent daily seizures were observed in case 7 with p.Arg1253Cys. He had an early‐onset age of 8 months and suffered from focal to bilateral tonic‐clonic seizures with a frequency of up to five times per day, which lasted for 3 years and then followed by complex partial seizures. The seizures in case 7 seem more severe than that in other cases. All the patients achieved seizure‐free with antiepileptic drugs.

Four cases, including two cases with p.Thr1119Ile (case 5 and 6), case 7 with p.Arg1253Cys, and case 11 with p.Asp1462Asn, displayed mild intellectual disability with difficulties in communication (Table 1). The motor development of all cases was normal. Brain MRI was normal in all cases.

Blood biochemical investigations of the cobalamin metabolism, including plasma vitamin B12, folate, and homocysteine levels, were conducted in eight of the 11 cases. The metabolic evaluations in these eight patients were essentially normal (Table 1).

### 3.7 *HCFC1* variants in validation cohort

To confirm the association between *HCFC1* variants and epilepsy, we collected additional 80 cases with partial epilepsy but without acquired causes from other clinical centers, including 31 trios and 49 singletons. Two variants (p.Thr1119Ile and p.Arg1253Cys) were recurrently identified in three additional patients each, including one homozygous p.Thr1119Ile and one homozygous p.Arg1253Cys (Table S3). Additionally, six novel hemizygous variants (c.1894A > G/p.Ile632Val, c.3563C > T/p.Ser1188Leu, c.3705T > G/p.His1235Gln, c.3734C > G/p.Ser1245Cys, c.3995C > T/p.Thr1332Met and c.4135G > A/p.Asp1379Asn) were identified in seven unrelated patients. Three variants, including c.1894A > G/p.Ile632Val, c.3705T > G/p.His1235Gln and c.3995C > T/p.Thr1332Met, were not present in general populations, and the other three variants were observed with a very low allele frequency of hemizygotes in gnomAD (Table S3). Five of these variants were located in the HCF‐1_PRO_ domain, and one variant p.Ile632Val in the basic domain.

The clinical and genetic features of the 13 patients with *HCFC1* variants from the validation cohort were summarized in Table S3. The onset age of seizures ranged from the first day to 9 years old, with a median onset age of 2 years. All of the cases had focal seizures or focal to bilateral tonic‐clonic seizures that can be controlled by monotherapy or polytherapy of antiepileptic drugs. The EEG recordings showed focal discharges. The two boys with p.Arg1253Cys both had very early‐onset age, frequent daily seizures and intellectual disability, sharing similar symptoms with case 7 with p.Arg1253Cys in our cohort. One boy with p.Ile632Val and one with p.His1235Gln had mild intellectual disability. All the cases had normal motor development.

### 3.8 Molecular sub‐regional implications of *HCFC1* variants

Previous studies have reported *HCFC1* variants in X‐linked cobalamin disorders^7^, ^8^ (CblX) and nonsyndromic intellectual disability without cobalamin abnormalities.^2^, ^5^, ^10^, ^21^ The present study identified novel *HCFC1* variants as a potential cause of benign partial epilepsy without cobalamin disorder. To understand the mechanism underlying phenotypic variations, we analysed genotype‐phenotype associations in all *HCFC1* variants with detailed phenotypes. Publications on *HCFC1* variants were retrieved from the PubMed database till Oct 2022. To date, 48 variants, including 44 missense variants, two splice‐site variants, a small deletion, and a 5′‐UTR variant, were identified in 98 individuals from 75 families. Their molecular locations and clinical data were listed in Table S4.

**FIGURE 5:**
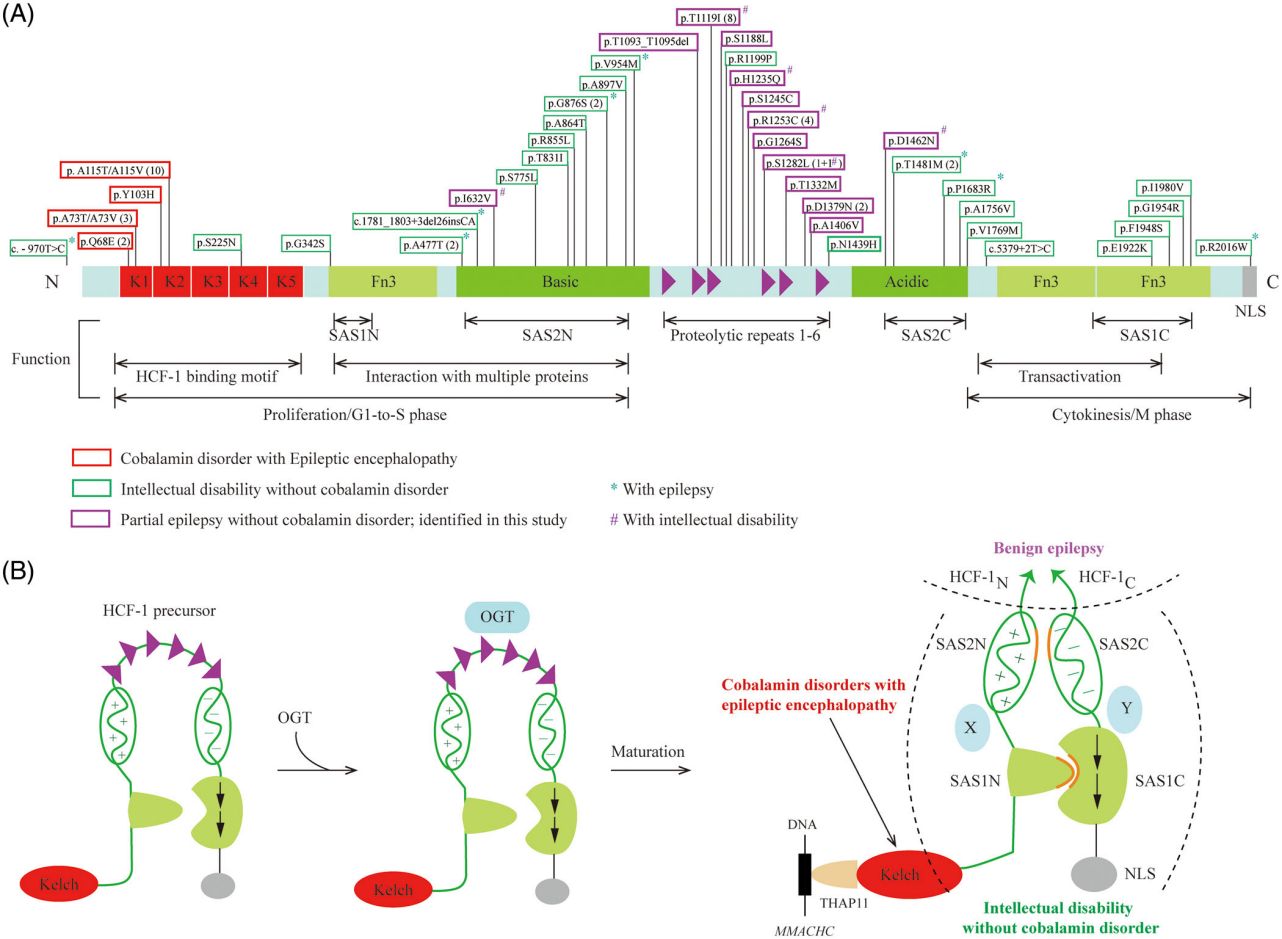
**The location and phenotypes of *HCFC1* variants. (A)** Location of the variants in HCF‐1 and their corresponding phenotypes. K1‐K5, the kelch domain; the basic domain; proteolytic repeats 1−6, the proteolysis domain; the acidic domain; Fn3, fibronectin 3 domains; and NLS, the nuclear localization signal domain. The major functions of each domain of HCF‐1 are indicated in the bottom. The number of affected unrelated cases is indicated in parentheses. **(B)** A proposed model of OGT‐induced HCF‐1 proteolytic maturation and the potential molecular sub‐regional effects of HCF‐1. The HCF‐1 precursor undergoes an unusual proteolytic maturation that is mediated by OGT. Cleavage occurs at the six centrally located HCF‐1_PRO_ repeats in a stochastic manner. The proteolytic HCF‐1_N_ and HCF‐1_C_ terminal fragments are noncovalently associated through two matched pairs of self‐association sequences (SAS1N‐SAS1C and SAS2N‐SAS2C). Variants within the proteolysis domain, which does not exist in the mature HCF‐1 complex, were associated with benign epilepsy. Variants in the kelch domain were associated with cobalamin disorders, and those in the self‐association complex were mainly associated with intellectual disability without cobalamin disorder.

Six missense variants were associated with cobalamin disorders, which were reported in 18 unrelated cases. All of the CblX‐associated variants were located within the K1 and K2 motifs of the kelch domain (Figure 5A). Twenty‐one missense variants were previously reported to be associated with intellectual disability without cobalamin disorders. The variants were located in regions from the K4 motif to the C terminus, of which majority (13/21, 61.9%) clustered in the basic and acidic domains, and only one in the proteolysis domain. In contrast, majority (11/13, 84.6%) of the variants identified in this study were located in the proteolysis domain and were associated with partial epilepsy without cobalamin disorder. The other two variants were located in the basic and acidic domains, respectively. The data suggested a molecular sub‐regional effect of *HCFC1* variants.

## 4 DISCUSSION

The present study identified seven hemizygous *HCFC*1 variants in 11 unrelated cases from a cohort of idiopathic partial epilepsies, and confirmed the finding in another cohort with additional 13 unrelated cases. None of patients had cobalamin disorders. Majority of the variants (11/13, 84.6%, in 91.7% cases) were located in the proteolysis domain. Functional studies demonstrated that the variants in the proteolysis domain potentially disrupted the proteolytic maturation of HCF‐1 and the inhibition of cell proliferation but did not affect *MMACHC* expression that was associated with cobalamin metabolism. Further genotype‐phenotype analysis demonstrated that variants within the proteolysis domain were associated with common partial epilepsy without cobalamin disorders, whereas those in the kelch domain were associated with cobalamin disorders featured by fatal epileptic encephalopathy, and those in the basic and acidic domains were mainly associated with intellectual disability without cobalamin disorders. The present study suggested that *HCFC1* was potentially a candidate pathogenic gene of common epilepsy with distinct mechanism of proteolysis dysfunction. The distinct difference between cobalamin disorders and idiopathic partial epilepsy in phenotype outcome and pathogenic mechanism, implied a clinical significance in early diagnosis and management.

Idiopathic partial (focal) epilepsy is a common group of self‐limited childhood epilepsies with infrequent focal seizures, benign course, and good prognosis, typically Rolandic epilepsy, that affects .2% of the population with an incidence of 10−20/100,000 children.^22^, ^23^, ^24^ Generally, the minor allele frequency (MAF) of genetic variants in general populations depends on phenotype prevalence^25^ that is related to phenotype severity. Variants that are associated with common diseases or mild phenotypes could be prevalent with low MAF in general populations, in contrast to those with rare diseases or severe phenotypes that usually are absent in general populations. In the present study, five of the variants within the proteolysis domain presented low frequencies (MAF < .0005) in general populations of gnomAD (Table 2), similar to some variants in genes associated with common partial epilepsy, such as those in *GRIN2A*,^26^ *DEPDC5* ^27^ and *UNC13B*.^12^ Incomplete penetrance is common in genetic disorders and challenges evaluation of the pathogenicity of variants. Functional studies were further performed to validate the damage effect of these variants. The *HCFC1* variants within the proteolysis domain disrupted the proteolytic processing with loss of growth suppression but did not affect *MMACHC* expression that was associated with cobalamin disorder and severe epileptic encephalopathy. The plain and mild functional consequences of the variants were consistent with the mild clinical phenotype with incomplete penetrance and low MAF in general populations.

The precursor HCF‐1 comprised of several conserved protein domains and undergoes an unusual proteolytic maturation that is mediated by OGT (Figure 5).^15^, ^16^ Cleavage occurs at the six centrally located HCF‐1_PRO_ repeats in a stochastic manner. The proteolytic HCF‐1_N_ and HCF‐1_C_ terminal fragments are non‐covalently associated with each other through two matched pairs of self‐association sequences (SAS1N‐SAS1C and SAS2N‐SAS2C),^28^ forming a mature HCF‐1 complex (Figure 5B). As a transcriptional coregulator, HCF‐1 functions as a molecular scaffold to link sequence‐specific transcription factors with enzymes capable of altering posttranslational modifications, forming a critical component of transcriptional regulatory network governing cell proliferation, cell‐cycle progression and cell viability.^29^

Proteolysis domain, which does not exist in the mature HCF‐1 complex, conducts proteolytic process that is necessary to separate and ensure the HCF‐1_N_ and HCF‐1_C_ functions in cell proliferation.^18^ The HCF‐1_N_ subunit promotes passage through the G1 phase of cell growth, and the HCF‐1_C_ subunit regulates proper exit from mitosis.^18^, ^19^, ^30^, ^31^ Previous experiments have demonstrated that variants, including large/small deletions of the repeats and point mutations in the cleavage and threonine regions (e.g., p.Glu1081Ala), disrupted the cleavage process.^3^, ^15^, ^16^ However, the variant‐associated clinical phenotypes have not been reported previously. The present study identified 11 variants in the proteolysis domain in 22 unrelated cases with partial epilepsy. The functional studies demonstrated that the variants in the canonical proteolytic sites in the threonine regions (p.Thr1093_Thr1095del and p.Thr1119Ile) disrupted the cleavage. The variant p.Arg1253Cys, located near the center of the proteolysis domain, resulted in obviously abnormal proteolytic fragments (Figure 2B). The three cleavage‐affected variants significantly affected the subsequent cell proliferation (Figure 3A). The other three variants between the repeats (p.Gly1264Ser, p.Ser1282Leu and p.Ala1406Val) did not cause visibly abnormal cleavage products but significantly caused loss of function in inhibition of cell proliferation (Figure 3A). These findings suggested that the proteolysis dysfunction of HCF‐1 and loss of growth suppression is potentially a novel pathogenic mechanism for epilepsy.

The mutant p.Arg1253Cys, which was located between the repeats, resulted in a distinct abnormal cleavage, suggesting the mutations in the cryptic regions beyond the canonical sites were also potentially affecting proteolytic process. Furthermore, mutant p.Arg1253Cys failed to localize on nucleus. The failure in nuclear localization was supposed to be caused by the loss of NLS in the HCF‐1_C_ subunit that may not be self‐associated with the new polypeptide after proteolysis. Clinically, the three cases with p.Arg1253Cys all presented severer manifestations with early‐onset age before 8 months and frequent daily seizures, potentially explained by the profound functional alterations of p.Arg1253Cys.

Previously reported variants clustered in the basic domain and the acidic domain were associated with intellectual disability without cobalamin disorder. In the present study, the patients with variants in the basic and acidic domains also had intellectual disability. Whereas, only six (27.3%) of the 22 patients with variants in the proteolysis domain had intellectual disability. The basic and acidic domains are core regions of SAS2N and SAS2C, which interact with each other to form a second association complex for the heterodimeric HCF‐1 (Figure 5B). The present study demonstrated that the variant (p.Asp1462Asn) in the acidic domain resulted in loss of inhibition of cell proliferation, consistent with the previous studies on variants in the basic and acidic domains.^5^ Cell proliferation leading to seizures is a common phenomenon. For example, variants in mTOR signaling pathway genes (e.g., *TSC1*, *TSC2*, *MTOR*, *DEPDC5*, *NPRL2* and *NPRL3*),^32^ which are involved in growth and cell proliferation, cause a spectrum of partial epilepsy syndromes with or without visible cortical structural abnormalities. Besides the dysfunction in regulating cell proliferation, variants in the basic and acidic domains potentially caused elongated axonal length of hippocampal neurons.^5^ Recently, a knock‐in zebrafish model harboring mutation in the *hcfc1a* gene (the *hcfc1a* ^co60/+^), one ortholog of *HCFC1*, increased neural precursor cells and expression of neuronal and glial markers, and subsequently led to abnormal swim patterns without speed deficits.^33^ These findings suggested the role of the basic and acidic domains in the neurodevelopment.

None of the patients with variants in the present study had clinical manifestation of cobalamin abnormalities. The functional study showed that variants in the proteolysis domain did not affect the expression of *MMACHC*. Further genotype‐phenotype analysis demonstrated that the variants associated with cobalamin disorders were all located within the K1 and K2 motifs of the kelch domain. The kelch domain of HCF‐1 forms an antiparallel β‐propeller structure that exerts protein‐protein interactions with transcription promoters.^34^, ^35^ Through binding with Thanatos‐associated protein 11 (THAP11),^29^, ^36^ HCF‐1 regulates expression of *MMACHC*,^4^, ^7^ which encodes a critical enzyme of the cobalamin pathway. Previous studies have showed that the CblX‐associated variants, which were within or adjacent to the β strands,^35^ severely reduced *MMACHC* expression in skin fibroblasts of patients with cobalamin disorders^7^ and in “cblX” mice with *Hcfc1^A115V/Y^*.^37^ In contrast, a K4‐located variant p.Ser225Asn, which was distant from β strands,^35^ showed a slight impact on the *MMACHC* expression^5^ and was not associated with cobalamin deficiency^2^ and there was a quantitative correlation between the degree of phenotypic penetrance and the *MMACHC* expression modulated by *HCFC1*.^4^

In conclusion, the present study indicated that *HCFC1* was potentially a candidate gene for common idiopathic partial epilepsy with distinct underlying mechanism of the proteolysis dysfunction and loss of growth suppression. The HCF‐1 domains played distinct functional roles and were associated with different clinical phenotypes, highlighting the sub‐molecular mechanism underlying phenotype heterogeneity. Disclosing the sub‐molecular effects and establishment of *HCFC1*‐epilepsy association will help the early diagnosis and management of patients with *HCFC1* variants.

## CONFLICT OF INTEREST STATEMENT

The authors declare that they have no competing interests.

## Supporting information

Supporting Information

Supporting Information

Supporting Information

Supporting Information

Supporting Information

Supporting Information

Supporting Information

## Data Availability

The data that support the findings of this study are available on request from the corresponding author. The data are not publicly available due to privacy or ethical restrictions.
