# Supplementary figures and images for: HCFC1 variants in the proteolysis domain are associated with X‐linked idiopathic partial epilepsy: Exploring the underlying mechanism
Source: Clin Transl Med. 2023 Jun 1;13(6):e1289. doi: 10.1002/ctm2.1289 (PMC10235798; doi:10.1002/ctm2.1289)

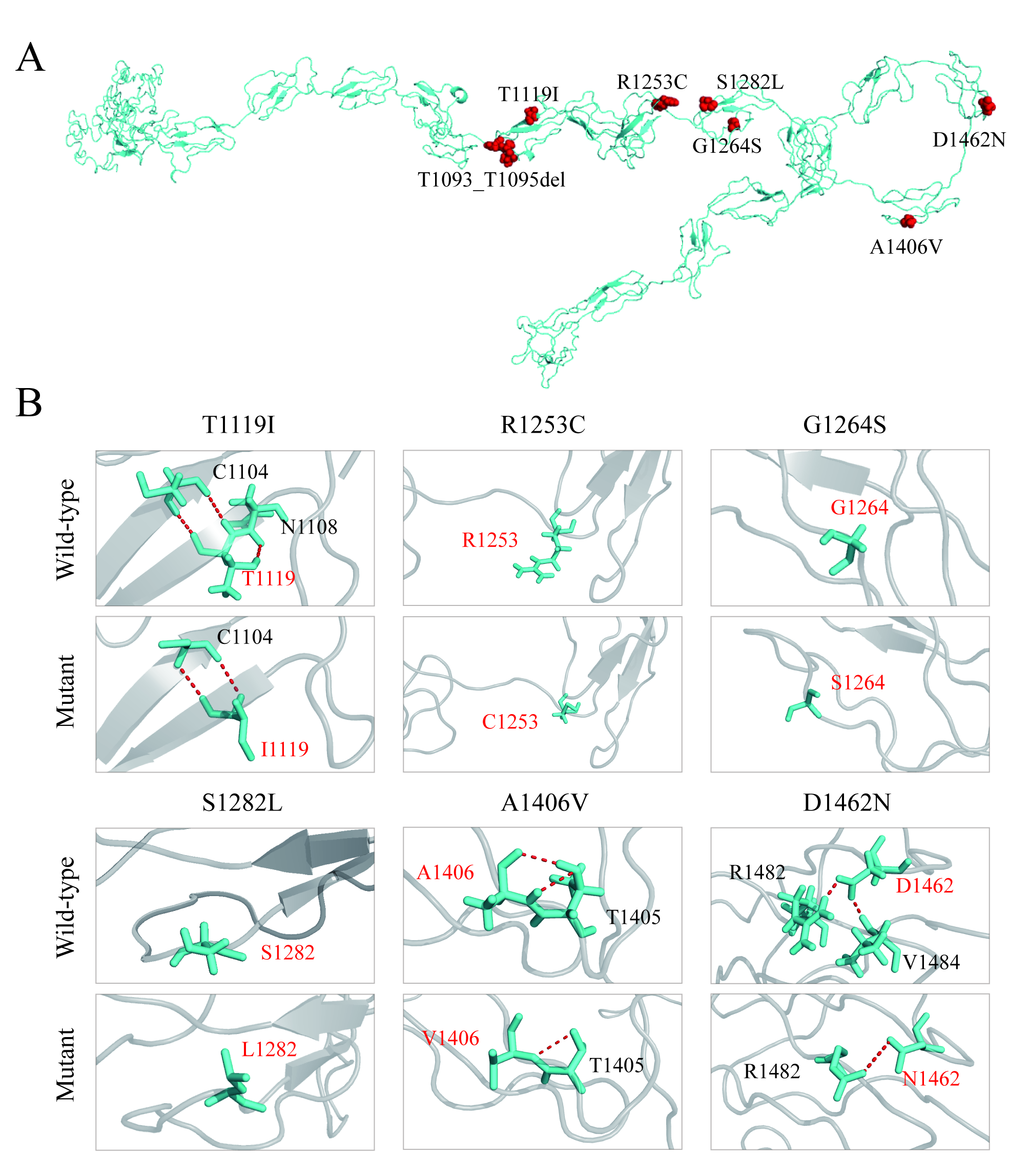

Supplement: Supplementary file 1 — Supporting Information [file CTM2-13-e1289-s006.tif]

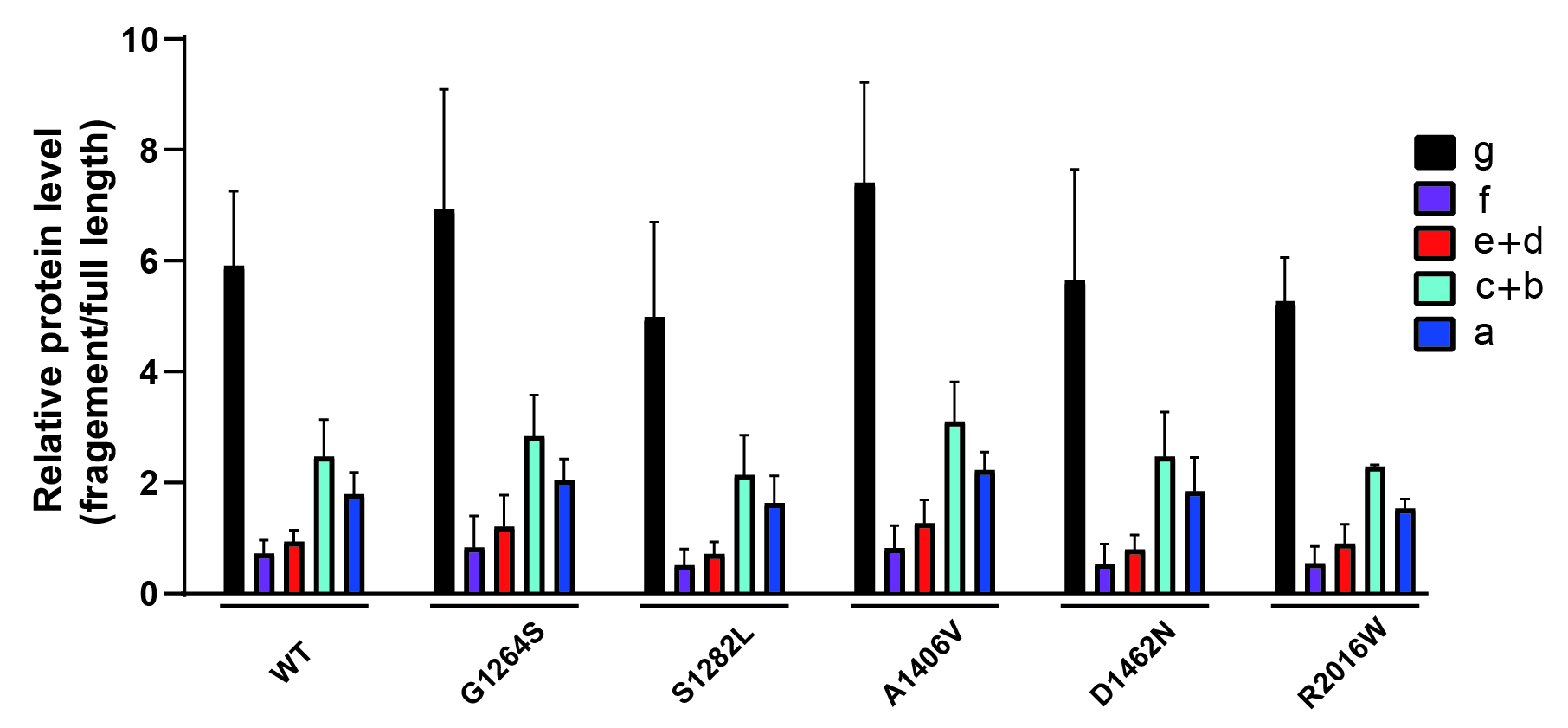

Supplement: Supplementary file 2 — Supporting Information [file CTM2-13-e1289-s007.tif]
