# Supplementary material for: HCFC1 variants in the proteolysis domain are associated with X‐linked idiopathic partial epilepsy: Exploring the underlying mechanism
Source: Clin Transl Med. 2023 Jun 1;13(6):e1289. doi: 10.1002/ctm2.1289 (PMC10235798; doi:10.1002/ctm2.1289)

**Supplementary Figure S3.1-S3.31 Mass spectrometry maps of peptide segments identified in protein band n.**

**Supplementary Figure S3.1**

**VVGWSGPVPR**

Location: aa22-31


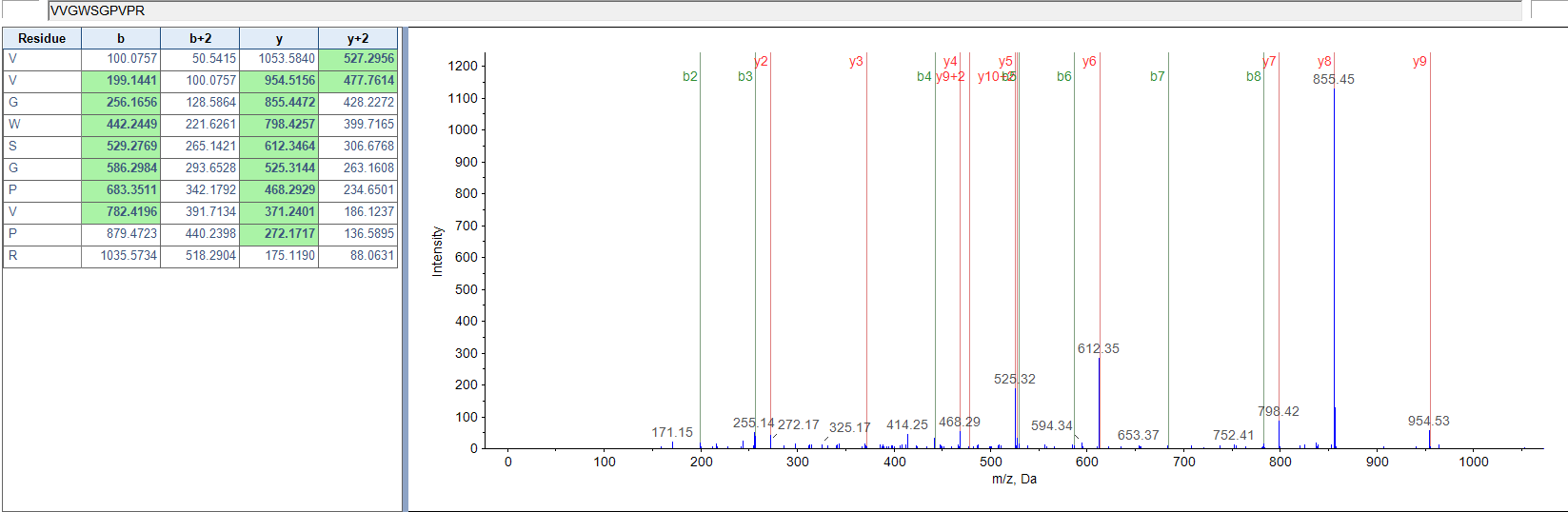


**Supplementary Figure S3.2**

**LLVFGGMVEYGK**

Location: aa94-105


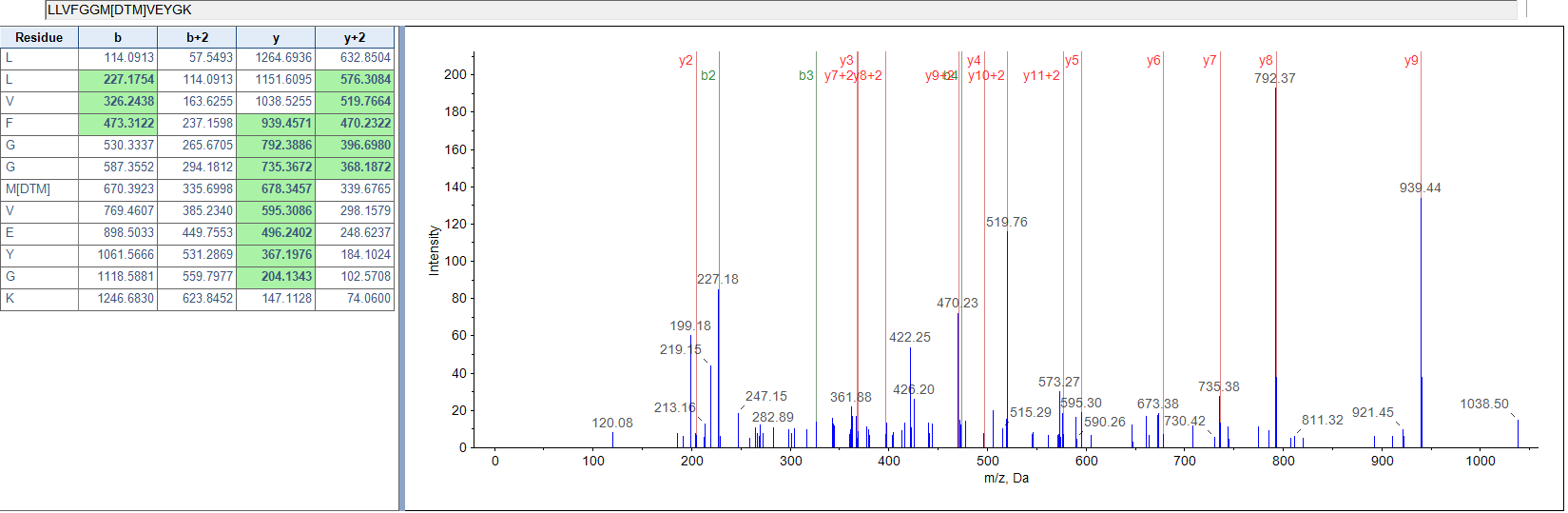


**Supplementary Figure S3.3**

**YSNDLYELQASR**

Location: aa106-117


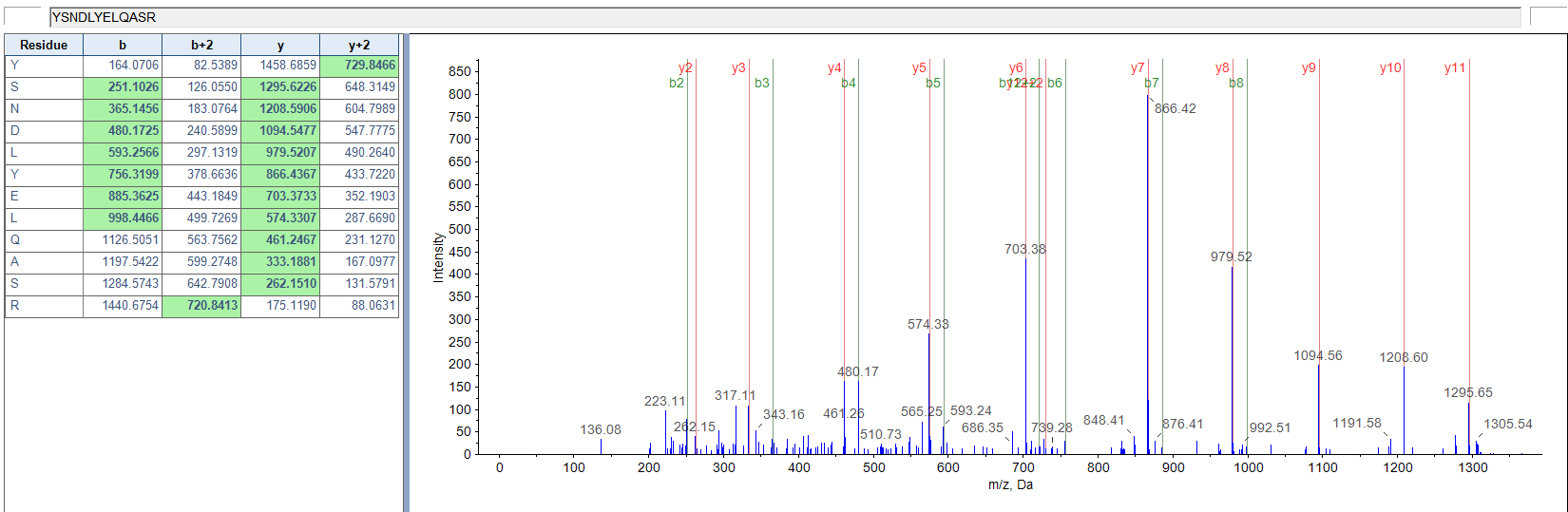


**Supplementary Figure S3.4**

**LGHSFSLVGNK**

Location: aa138-148


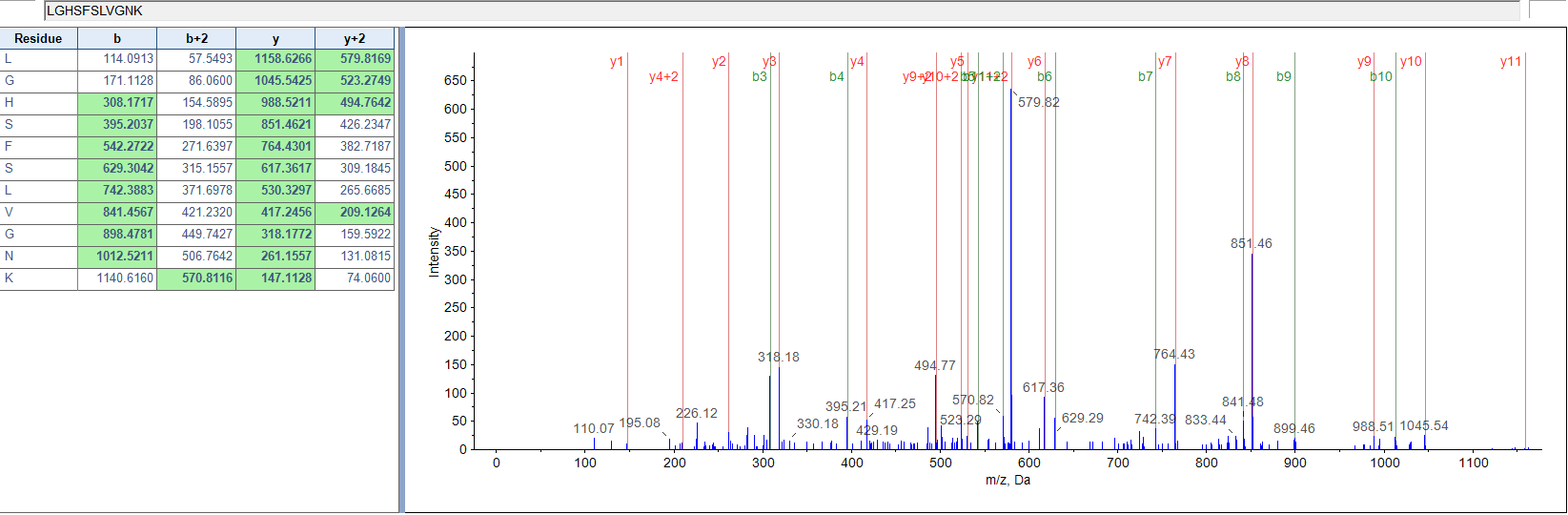


**Supplementary Figure S3.5**

**CYLFGGLANDSEDPKNNIPR**

Location: aa149-168

**
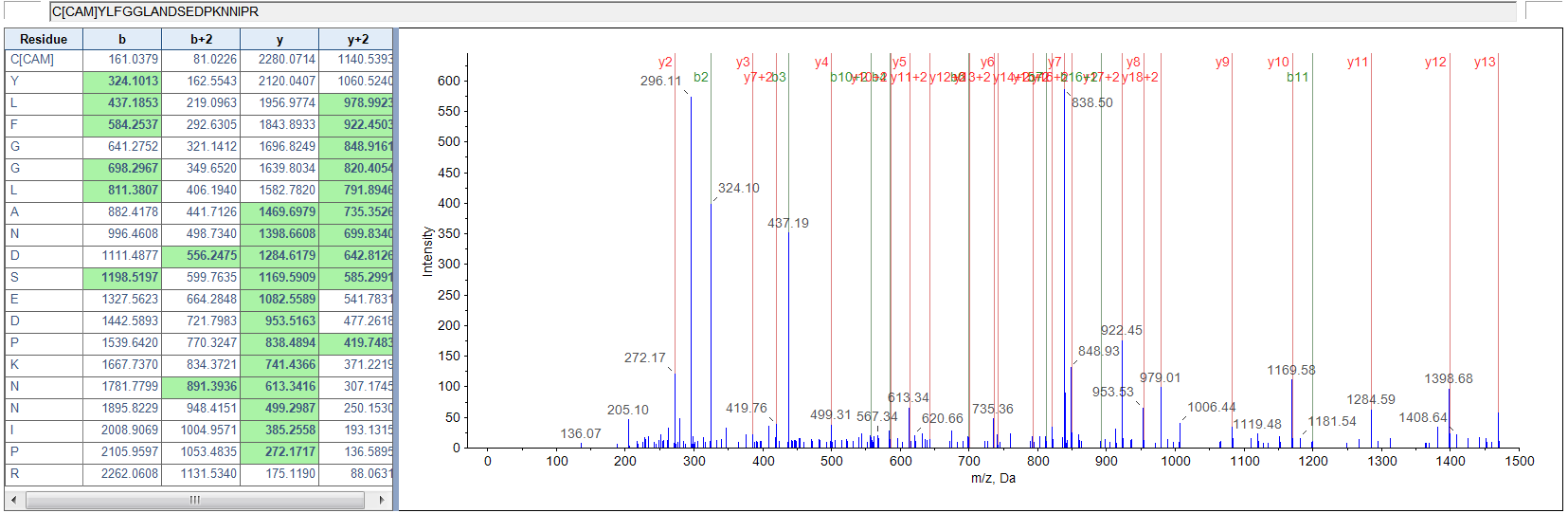
**

**Supplementary Figure S3.6**

**ESHTAVVYTEK**

Location: aa201-211

**
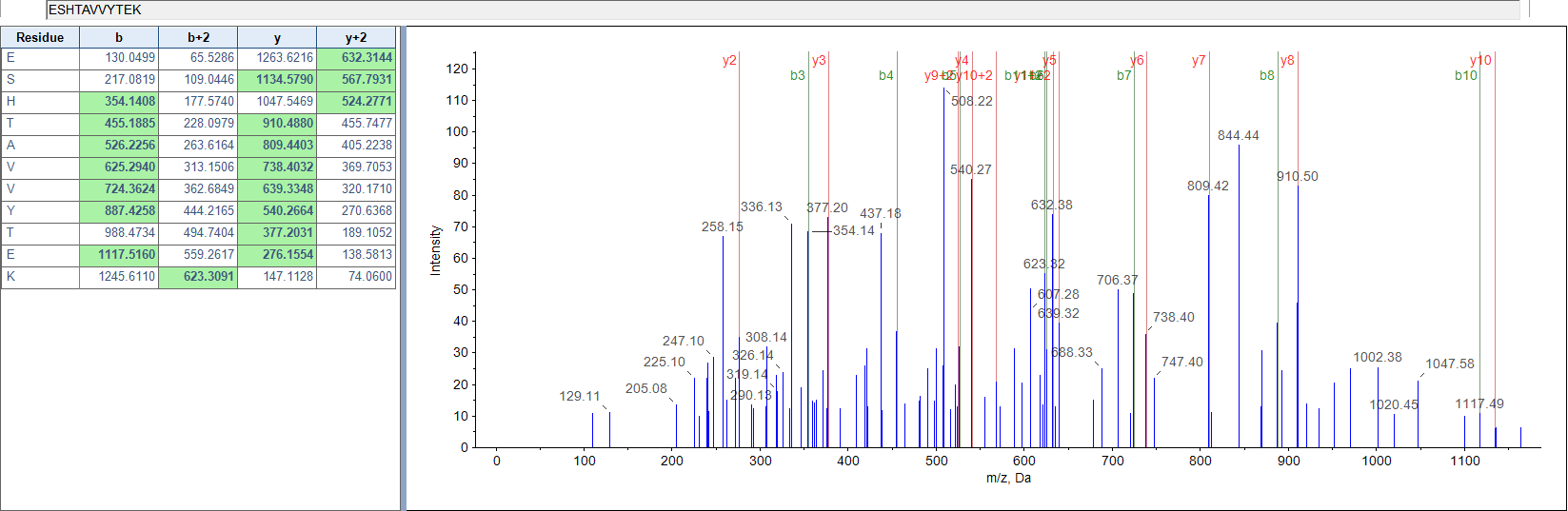
**

**Supplementary Figure S3.7**

**ESHTAVVYTEKDNKK**

Location: aa201-215


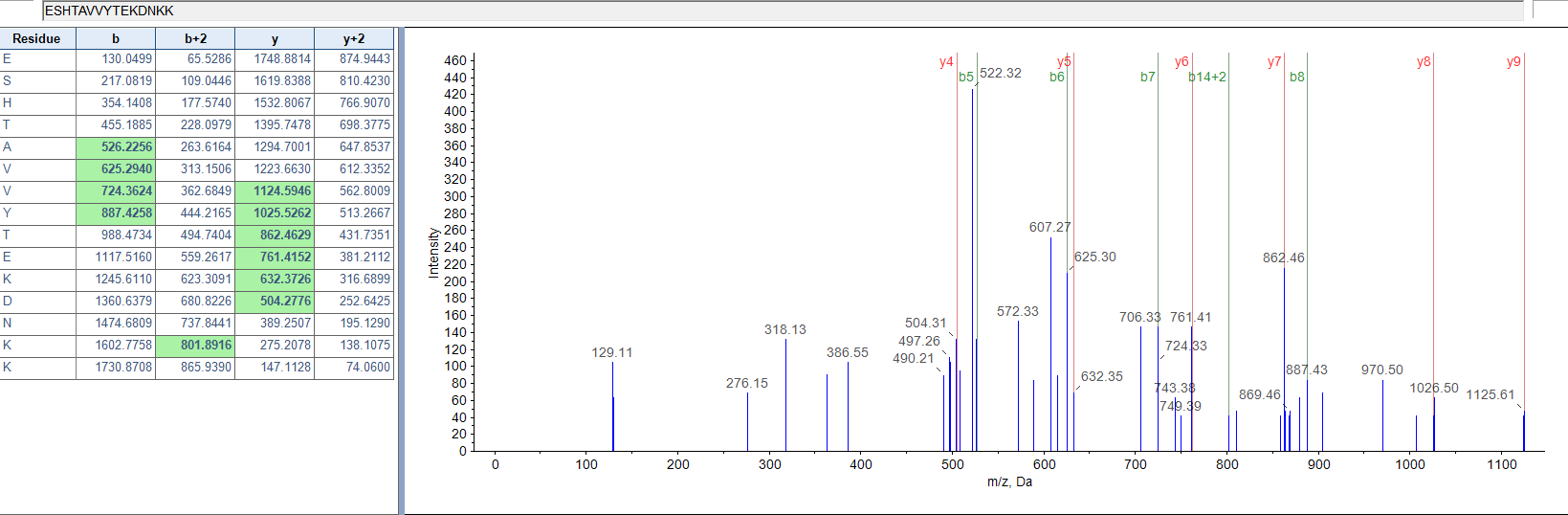


**Supplementary Figure S3.8**

**LVIYGGMSGCR**

Location: aa218-228


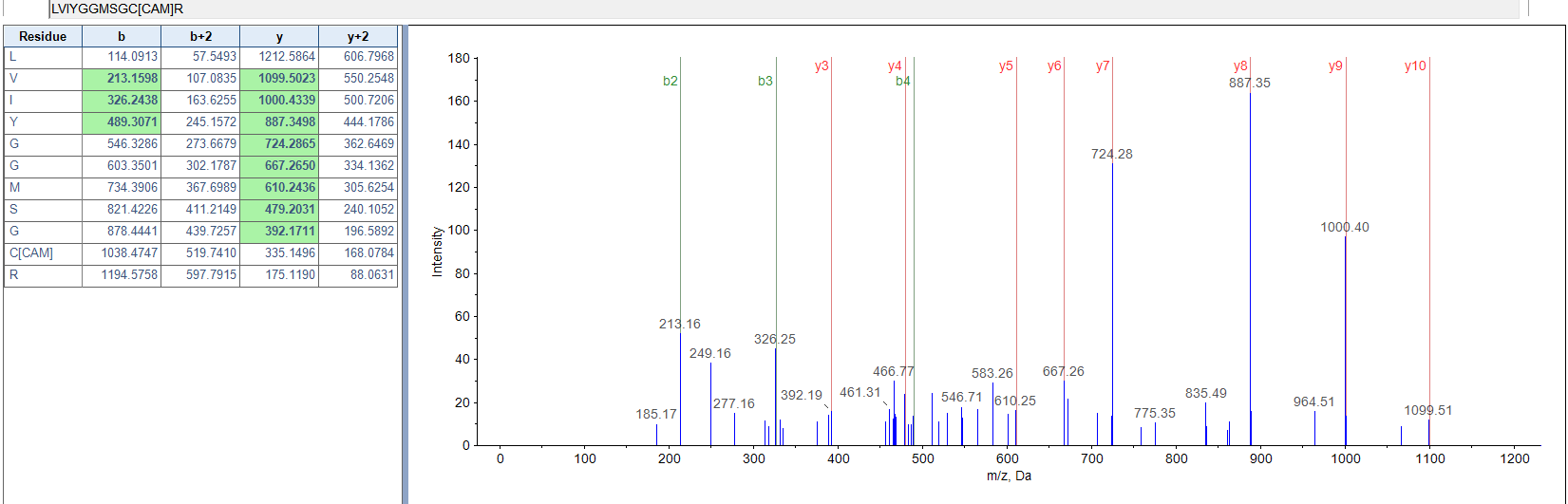


**Supplementary Figure S3.9**

**SLHSATTIGNK**

Location: aa256-266


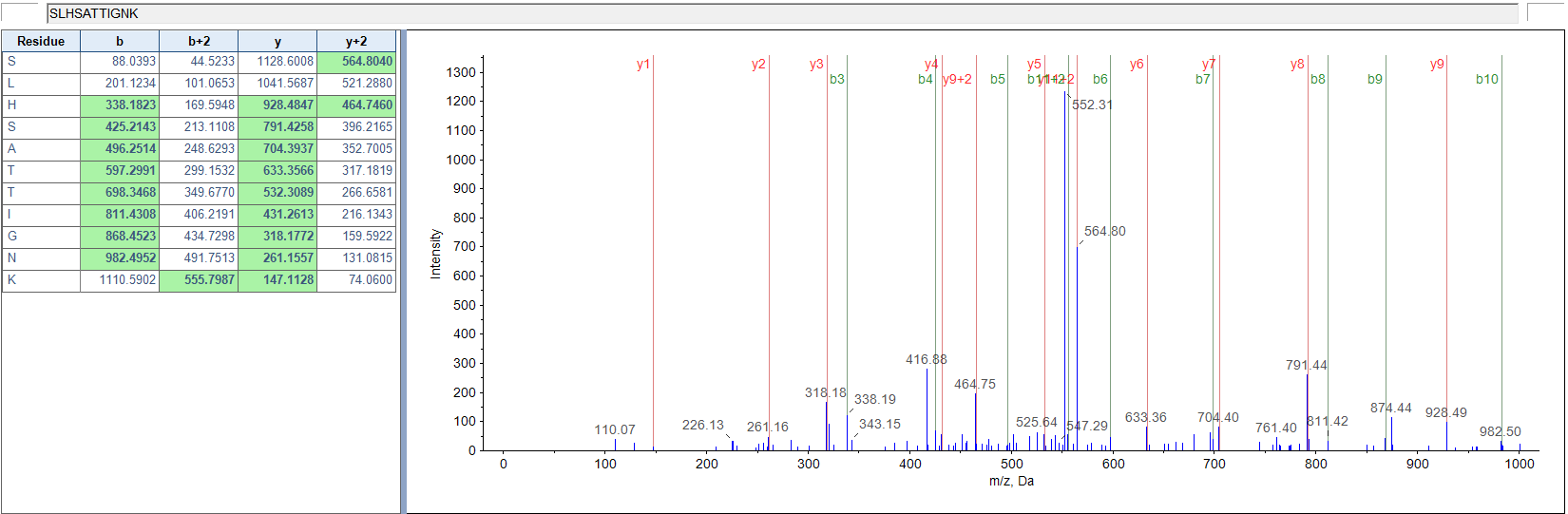


**Supplementary Figure S3.10**

**AGHCAVAINTR**

Location: aa323-333


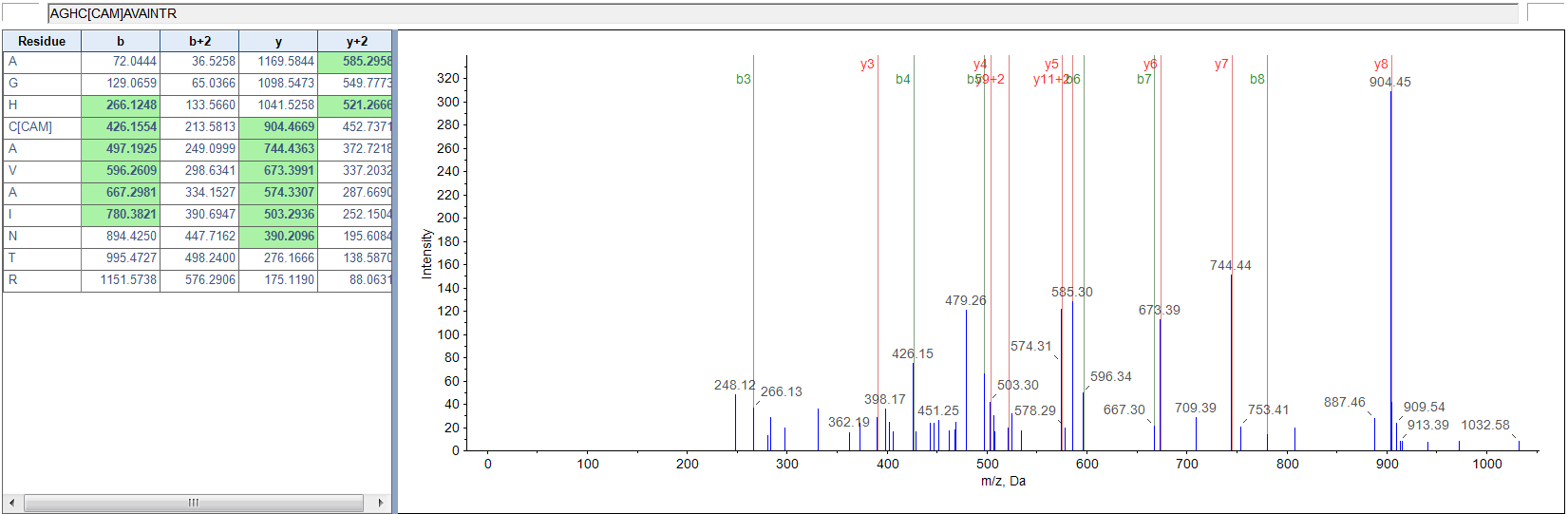


**Supplementary Figure S3.11**

**KAWNNQVCCK**

Location: aa345-354


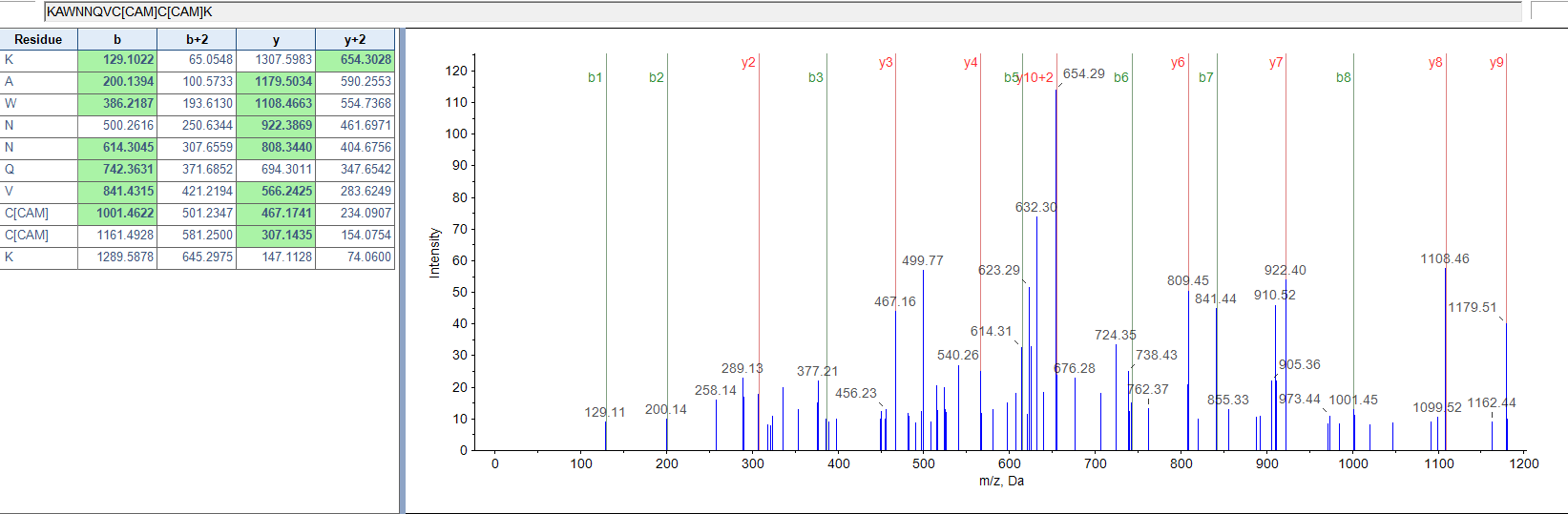


**Supplementary Figure S3.12**

**AWNNQVCCK**

Location: aa346-354

**
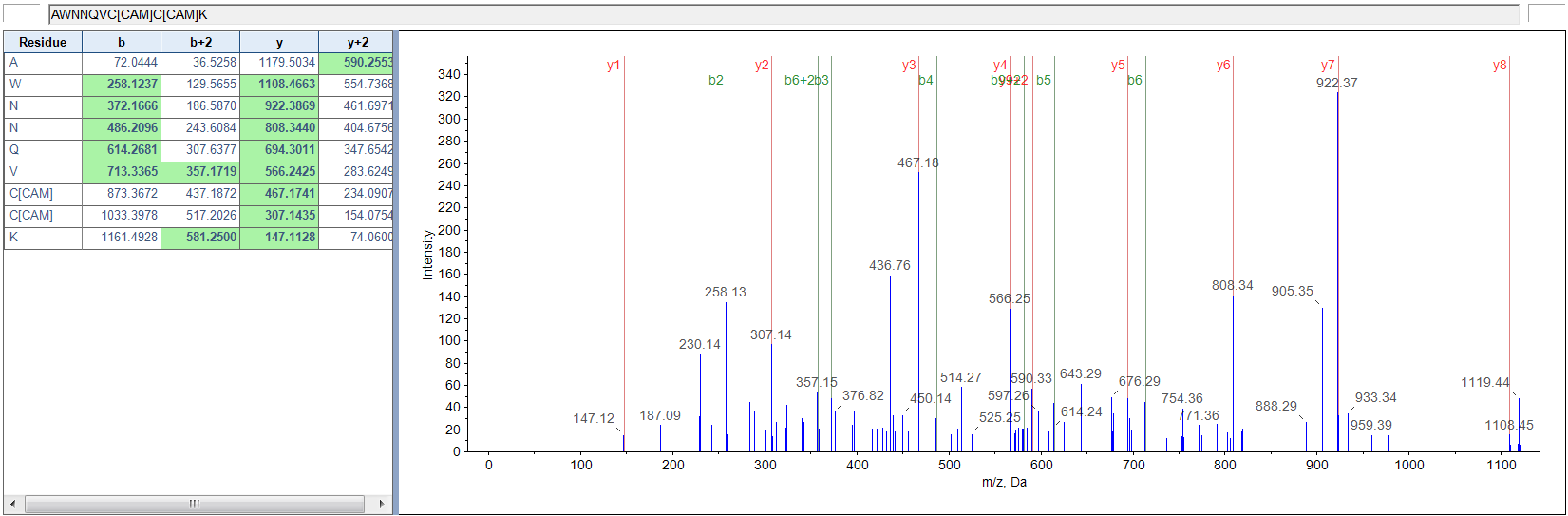
**

**Supplementary Figure S3.13**

**DLWYLETEKPPPPAR**

Location: aa355-369


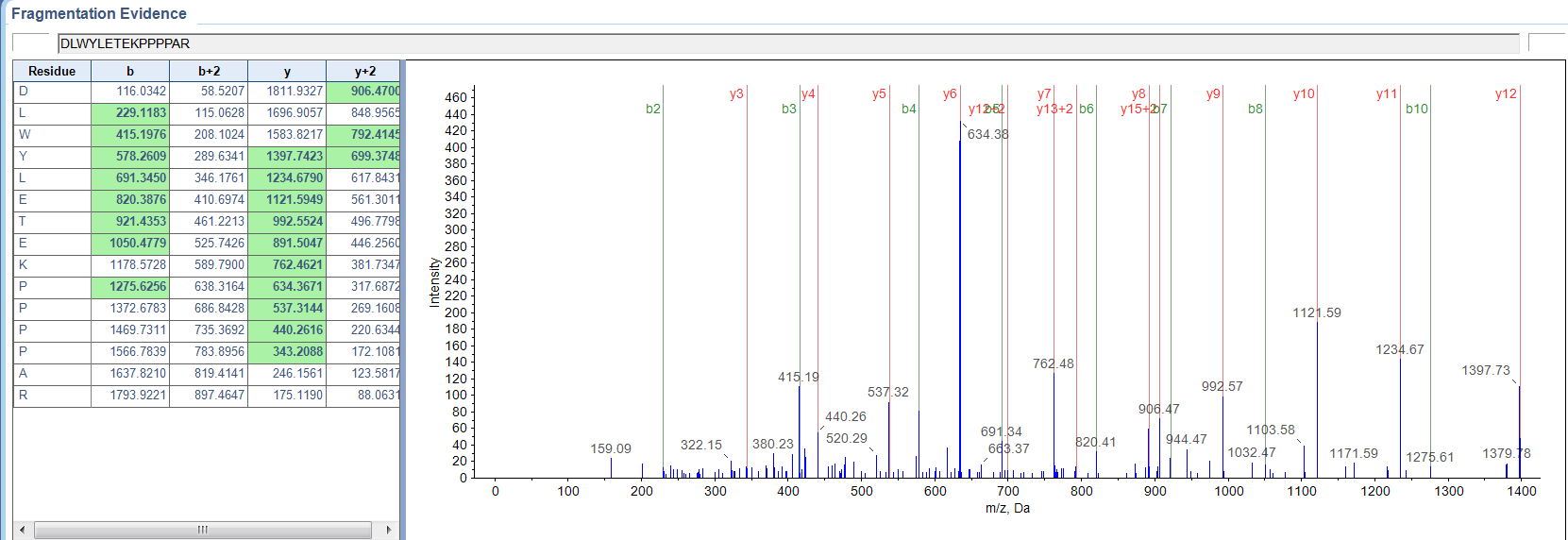


**Supplementary Figure S3.14**

**TQGVPAVLK**

Location: aa480-488

**
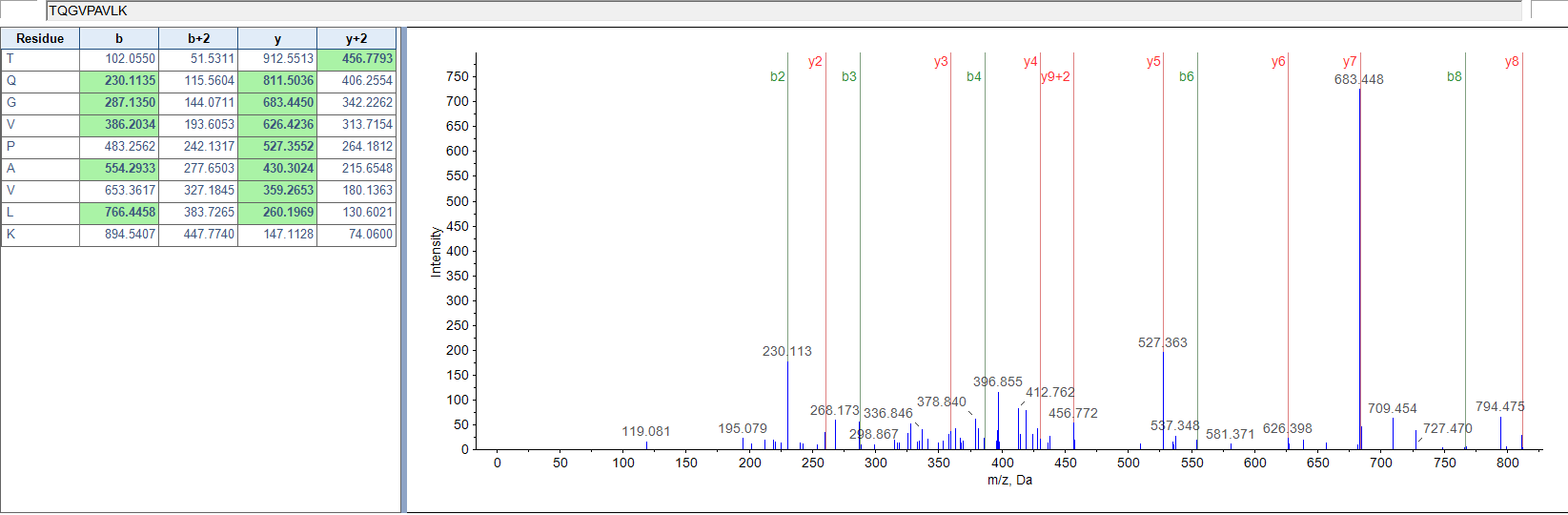
**

**Supplementary Figure S3.15**

**VTGPQATTGTPLVTMRPASQAGK**

Location: aa489-511

**
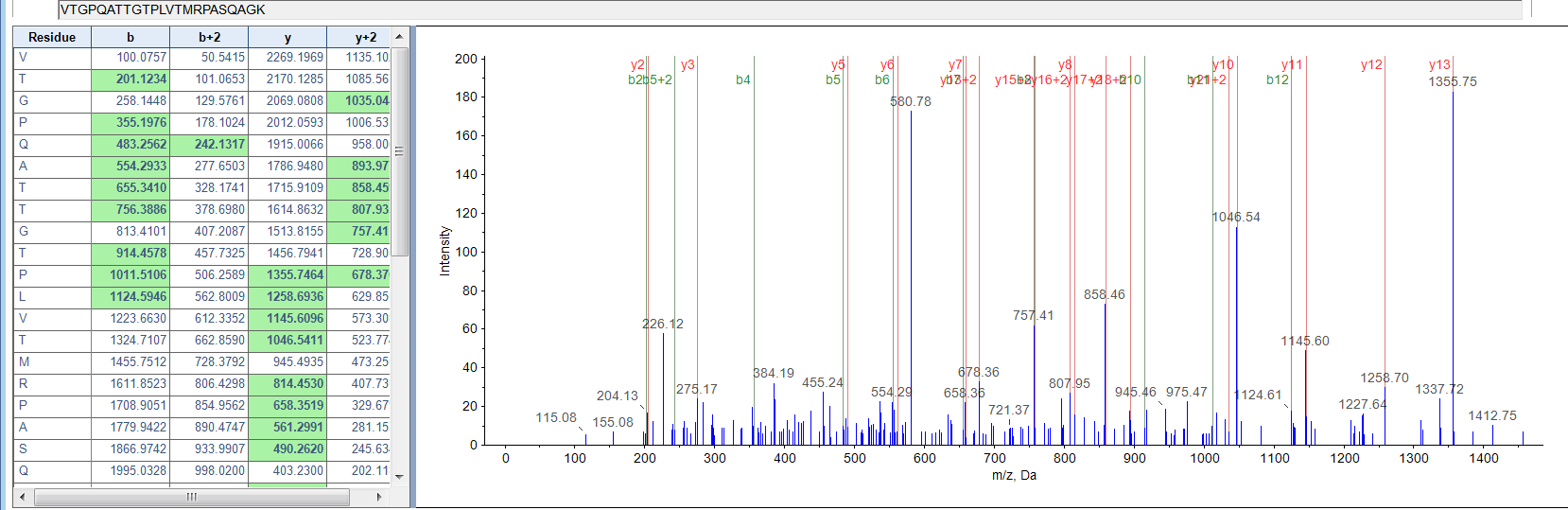
**

**Supplementary Figure S3.16**

**APVTVTSLPAGVR**

Location: aa512-524


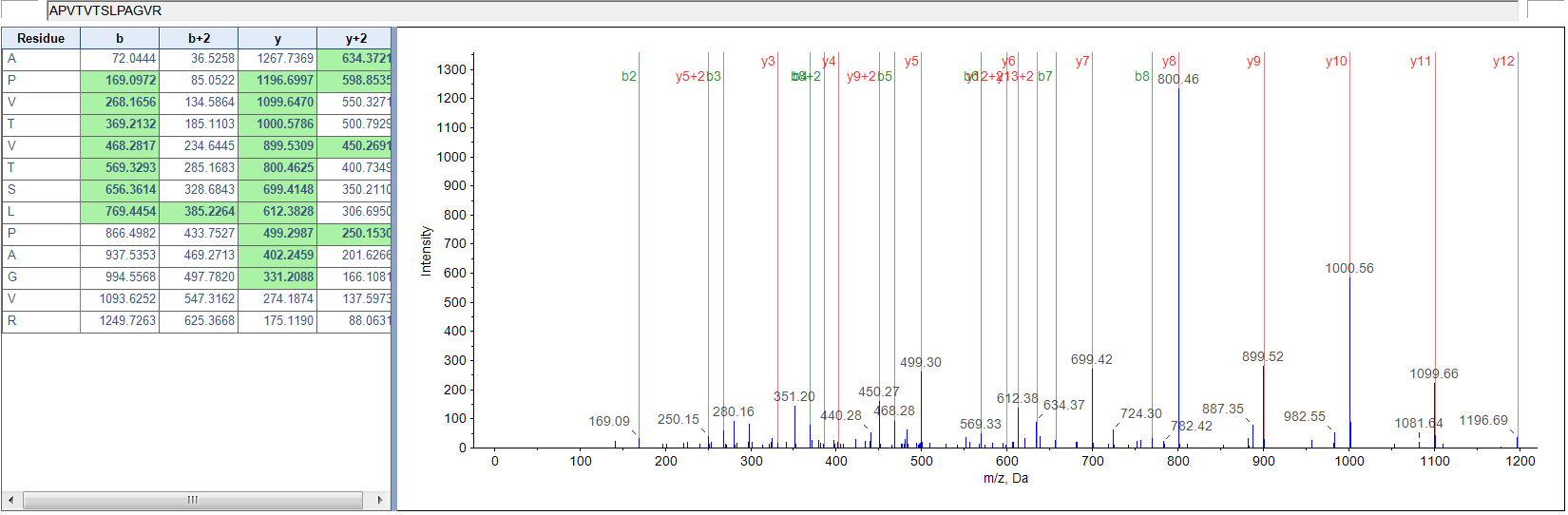


**Supplementary Figure S3.17**

**TMAVTPGTTTLPATVK**

Location: aa579-594


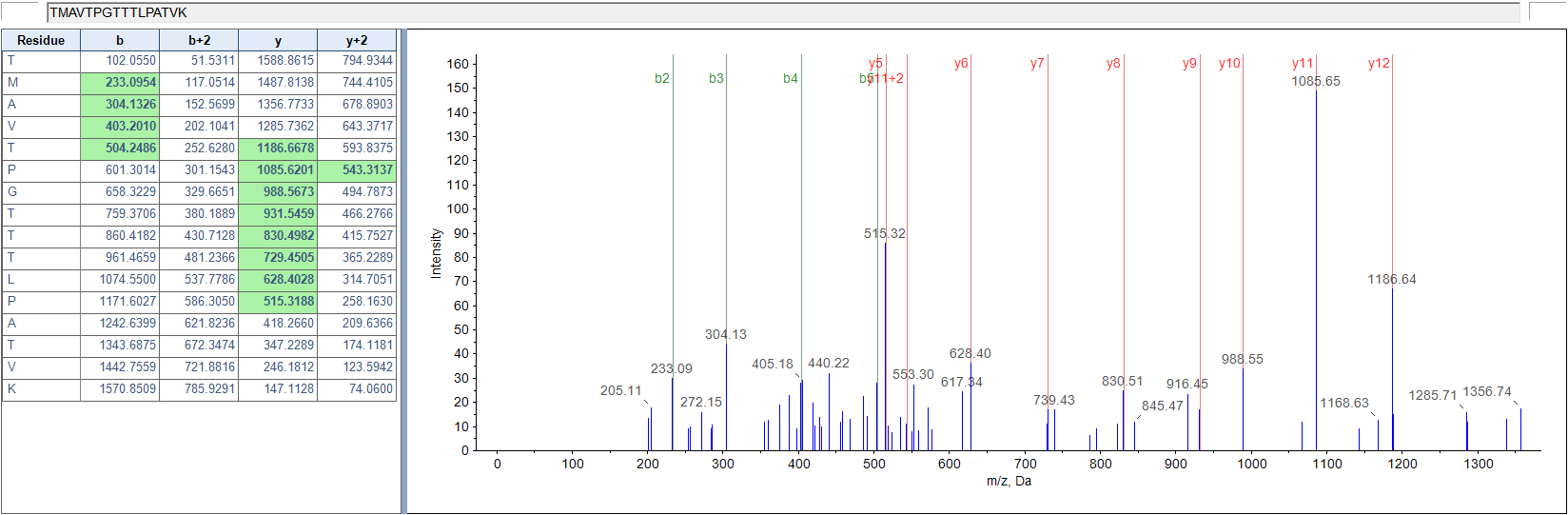


**Supplementary Figure S3.18**

**VASSPVMVSNPATR**

Location: aa595-608


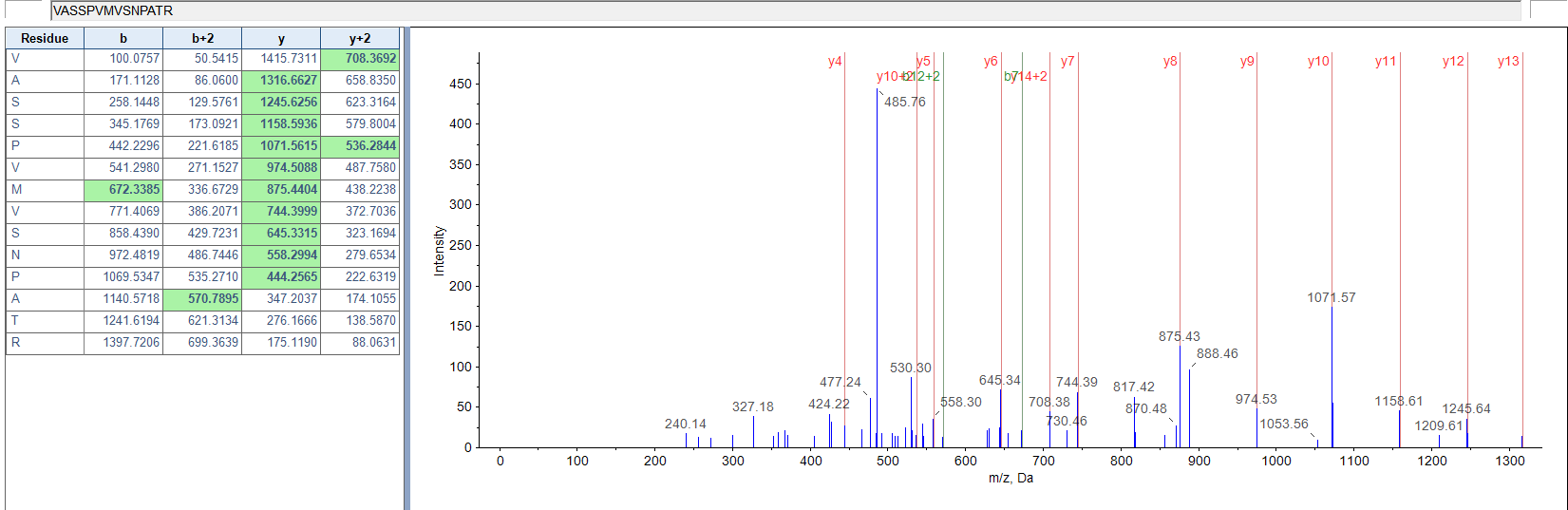


**Supplementary Figure S3.19**

**SGTVTVAQQAQVVTTVVGGVTK**

Location: aa638-659


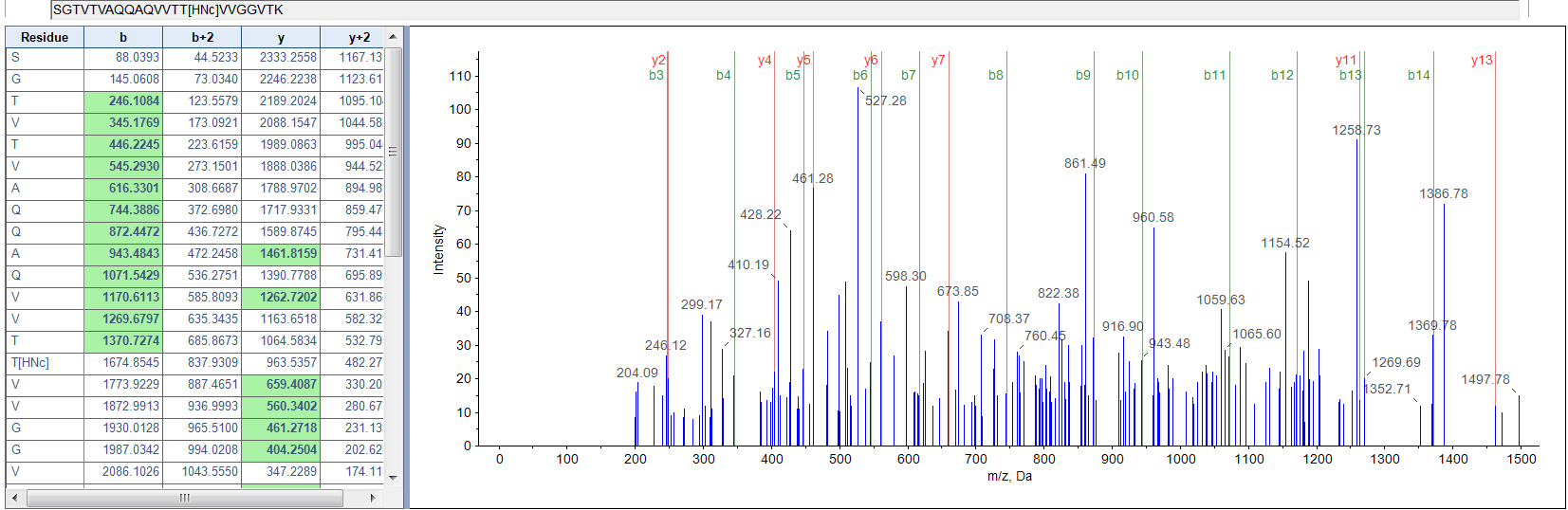


**Supplementary Figure S3.20**

**SPISVPGGSALISNLGK**

Location: aa666-682


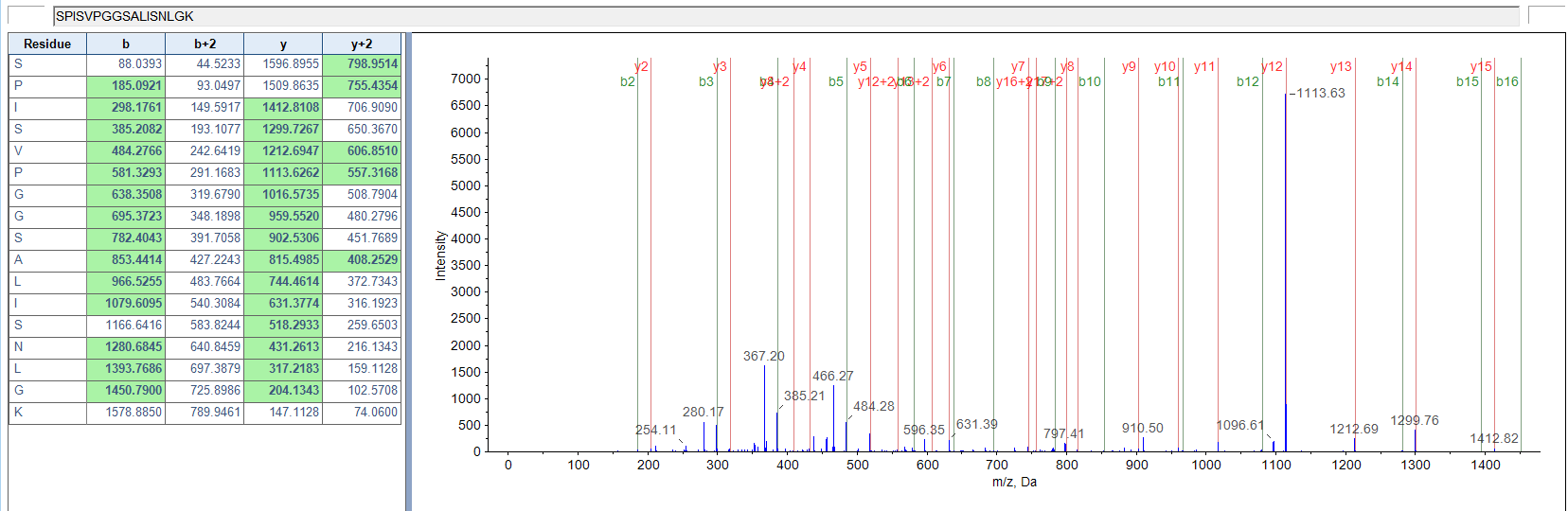


**Supplementary Figure S3.21**

**GPLPAGTILK**

Location: aa714-723


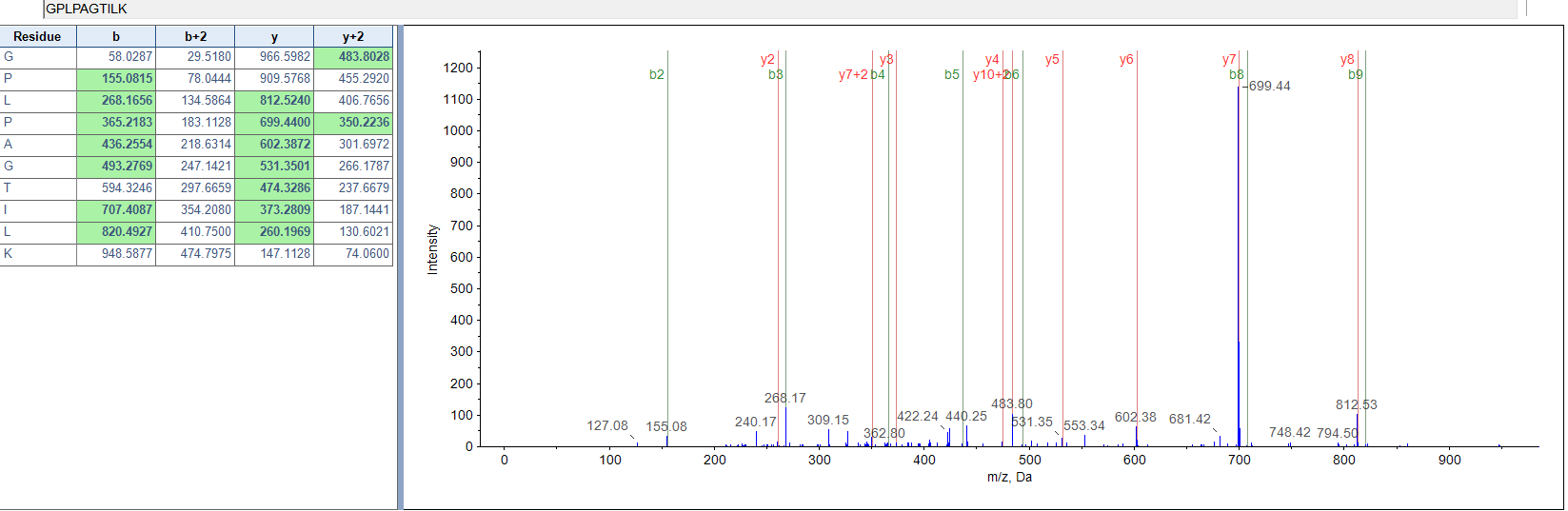


**Supplementary Figure S3.22**

**SPITIITTK**

Location: aa794-802


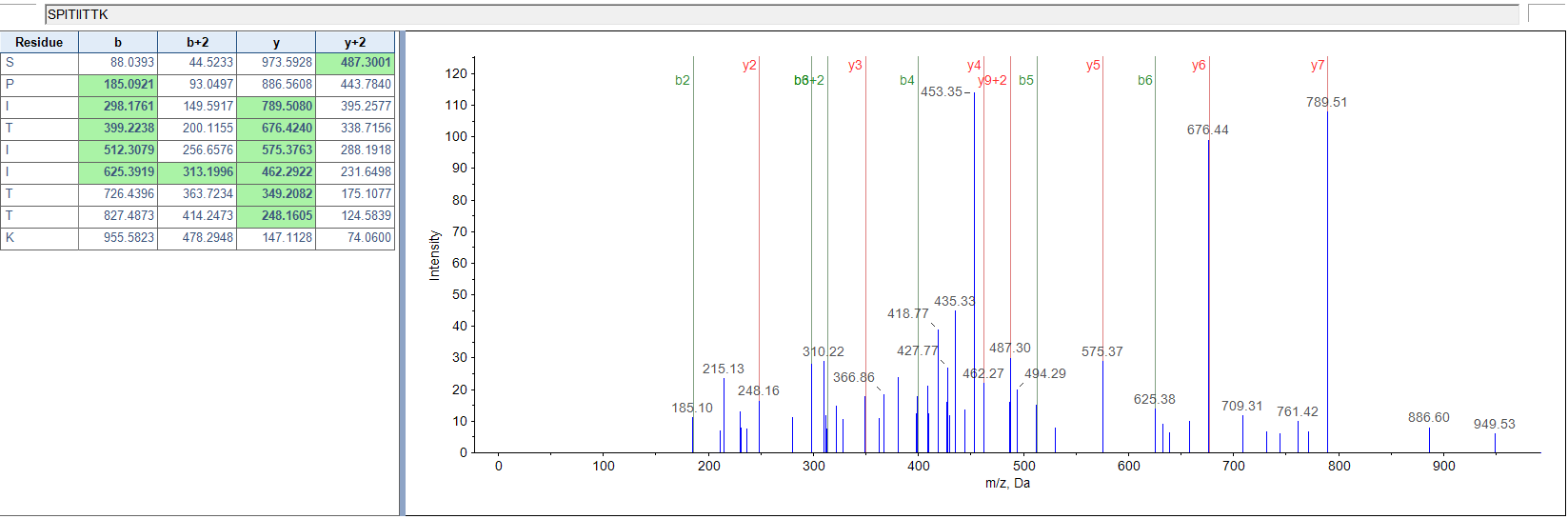


**Supplementary Figure S3.23**

**IATGHGQQGVTQVVLK**

Location: aa821-836


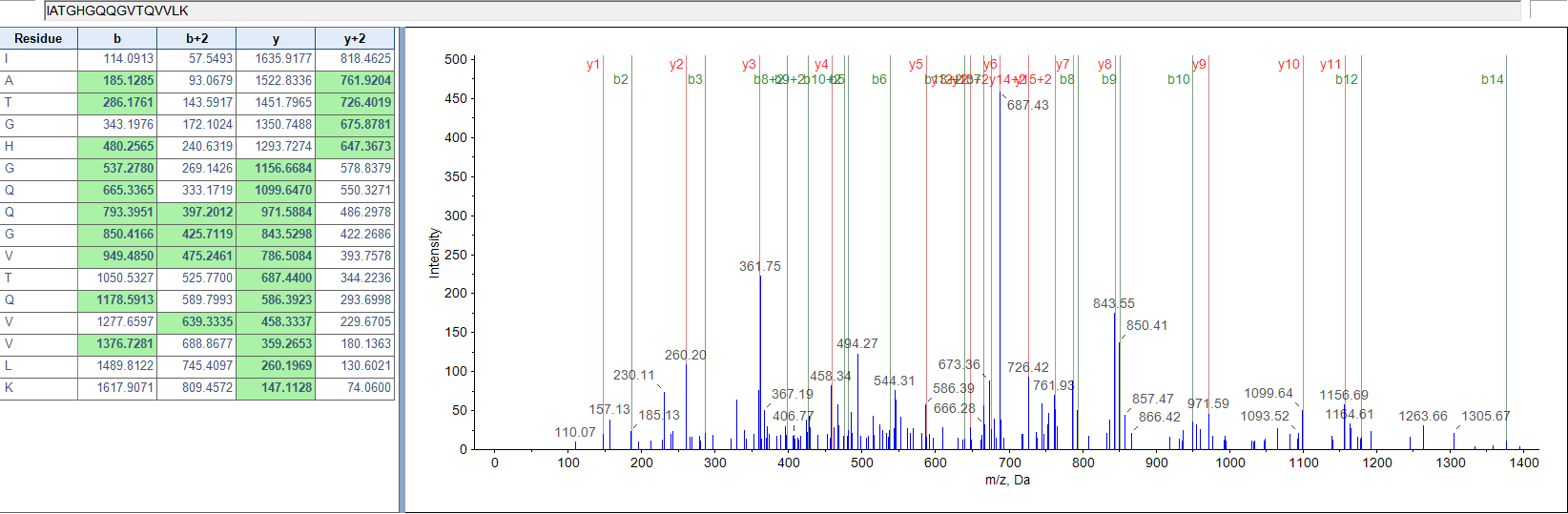


**Supplementary Figure S3.24**

**GAPGQPGTILR**

Location: aa837-847


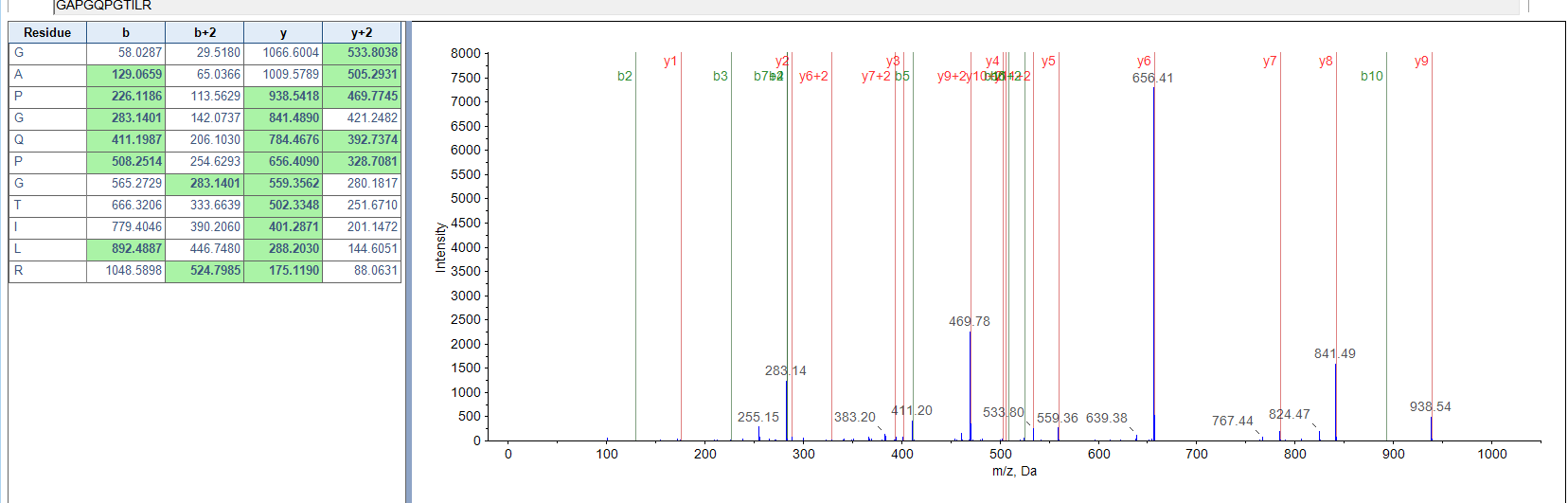


**Supplementary Figure S3.25**

**RTVPMGGVRLVTPVTVSAVKPAVTTLVVK**

Location: aa847-875


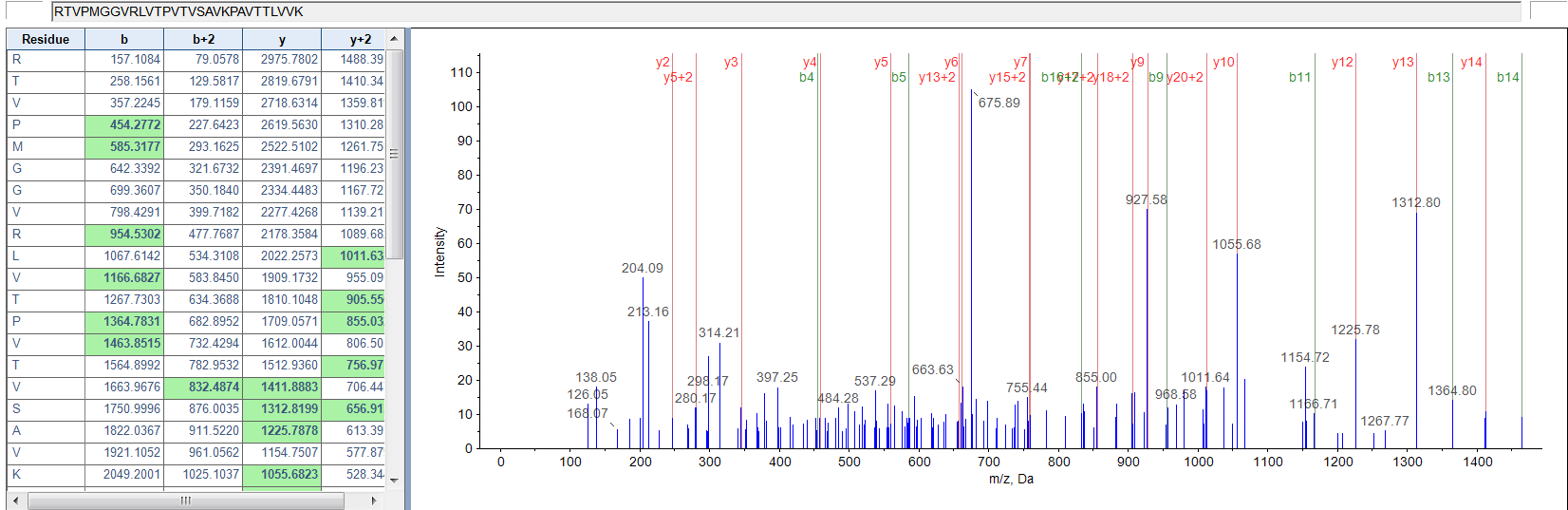


**Supplementary Figure S3.26**

**LVTPVTVSAVKPAVTTLVVK**

Location: aa856-875


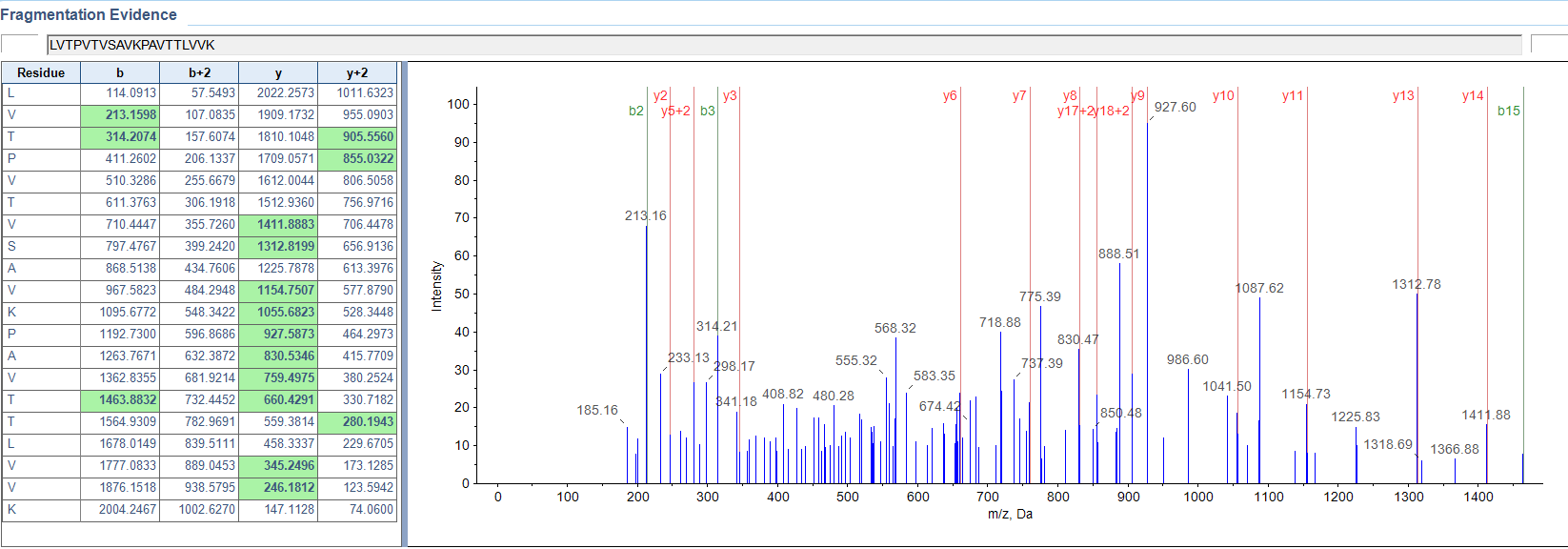


**Supplementary Figure S3.27**

**QEAAASLVTSTVGQQNGSVVR**

Location: aa1053-1073


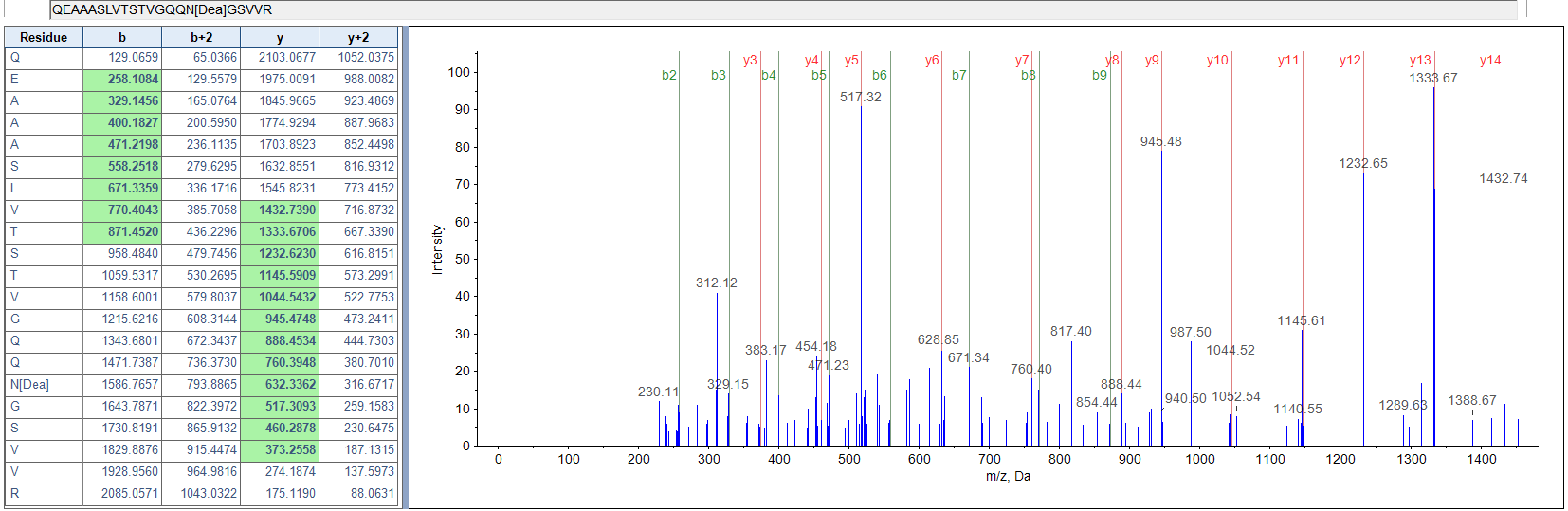


**Supplementary Figure S3.28**

**RACAAGTPAVIR**

Location: aa1137-1148


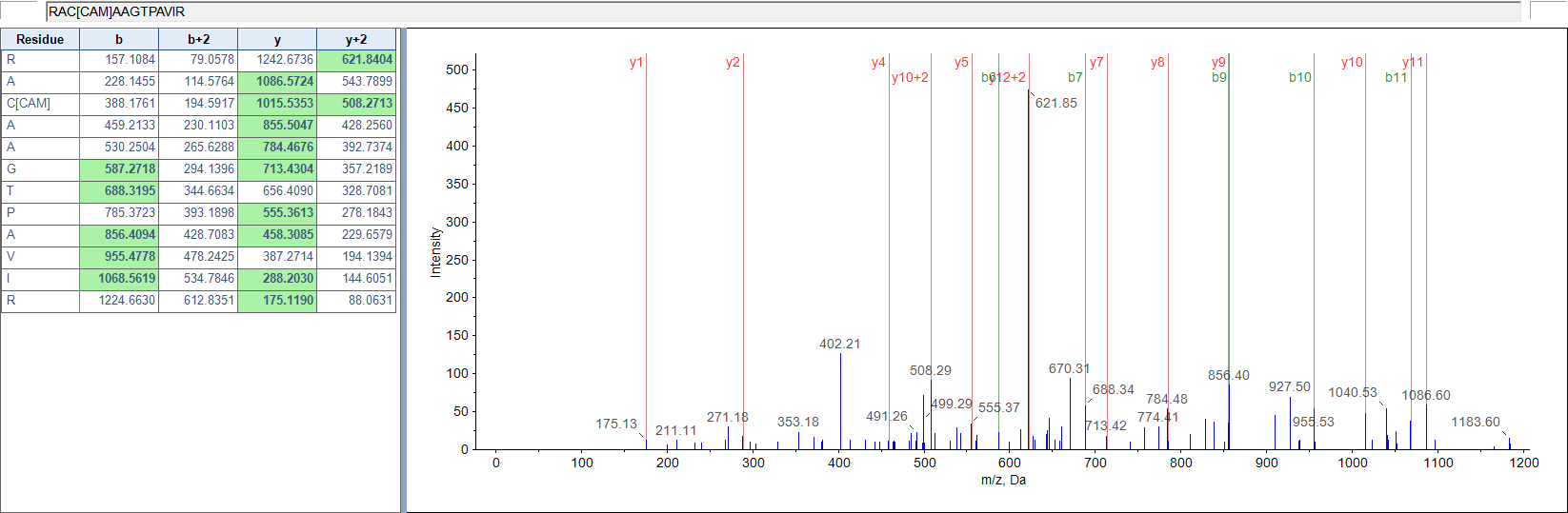


**Supplementary Figure S3.29**

**ACAAGTPAVIR**

Location: aa1138-1148


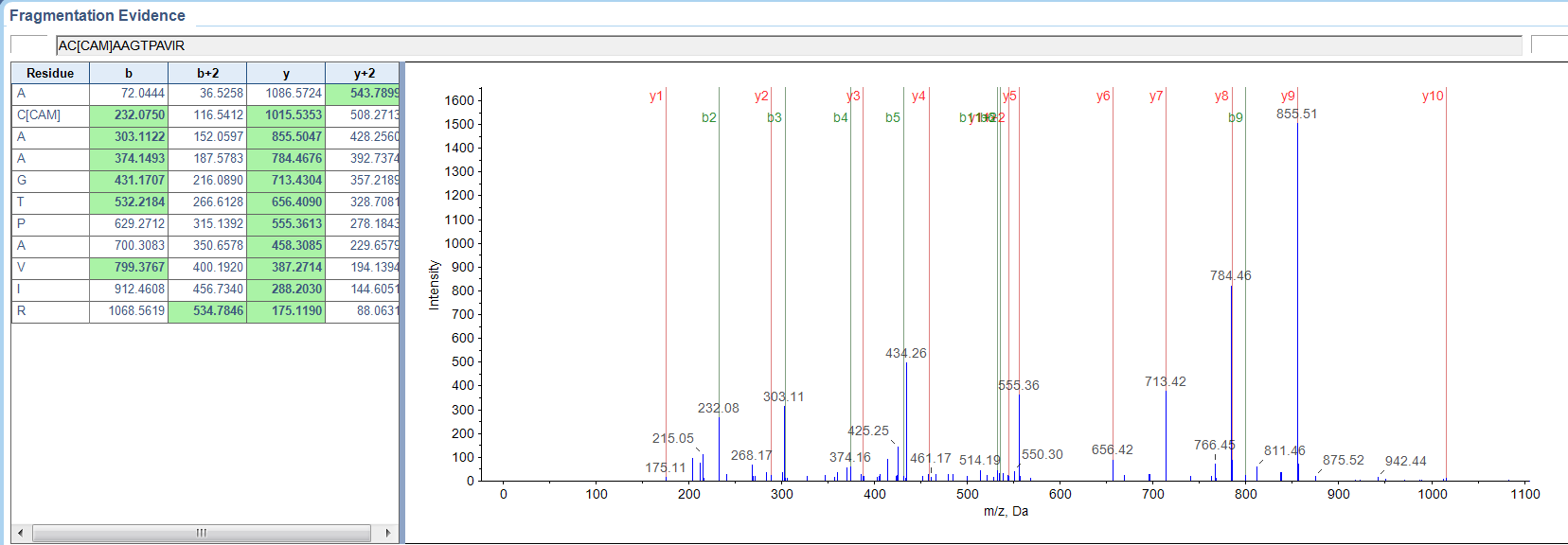


**Supplementary Figure S3.30**

**ISVATGALEAAQGSK**

Location: aa1149-1163


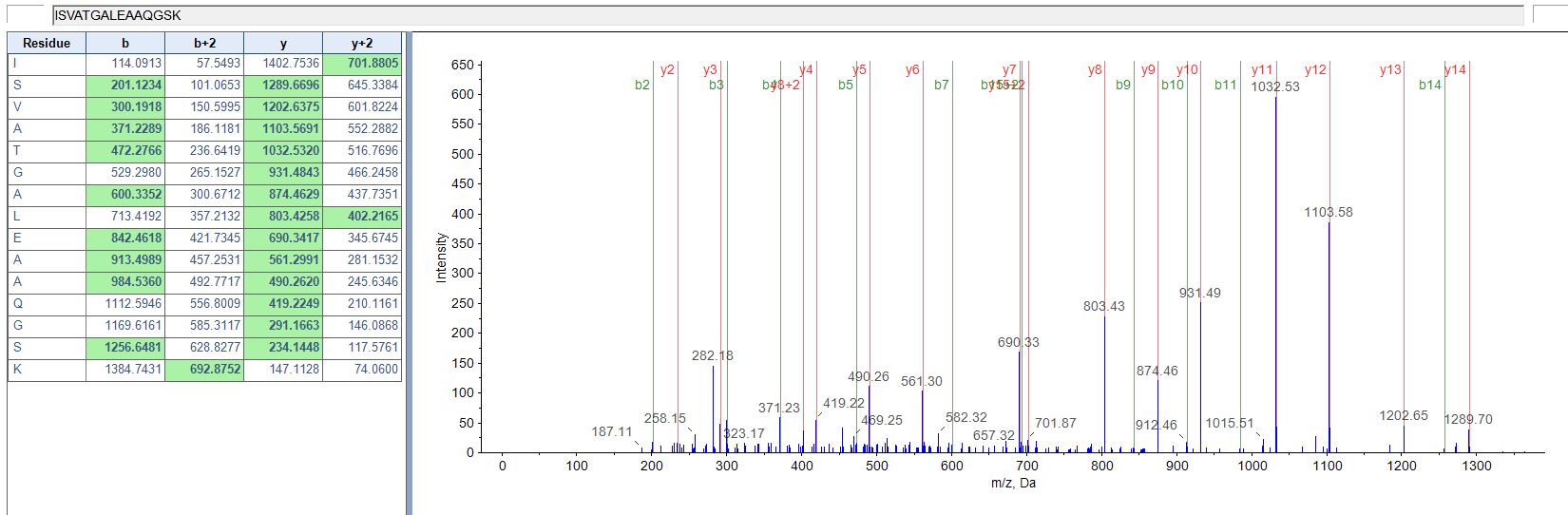


**Supplementary Figure S3.31**

**SPAFVQLAPLSSK**

Location: aa1205-1217


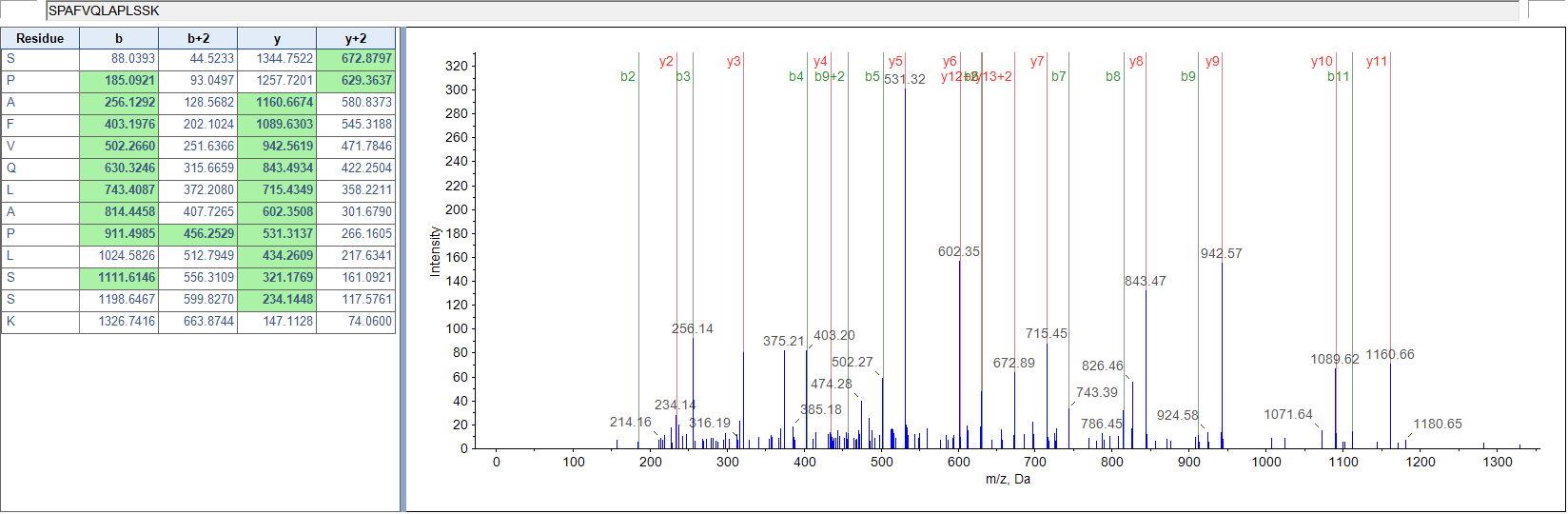

Supplement: Supplementary file 3 — Supporting Information [file CTM2-13-e1289-s001.docx]
