# Supplementary material for: HCFC1 variants in the proteolysis domain are associated with X‐linked idiopathic partial epilepsy: Exploring the underlying mechanism
Source: Clin Transl Med. 2023 Jun 1;13(6):e1289. doi: 10.1002/ctm2.1289 (PMC10235798; doi:10.1002/ctm2.1289)
Supplement: Supplementary file 4 — Supporting Information [file CTM2-13-e1289-s003.docx]

**Supplemental Table 2. Bioinformatics analysis of the *HCFC1* variants.**

| Variants | MAF | MAF-EAS | ACMG | | SIFT | LRT | MutationTaster | Fathmm_MKL | DANN | M-CAP | CADD | GenoCanyon | GERP++ | phyloP |
| --- | --- | --- | --- | --- | --- | --- | --- | --- | --- | --- | --- | --- | --- | --- |
|  |  |  | **Scoring** | **Rating** |  |  |  |  |  |  |  |  |  |  |
| c.3277_3285delACCGCCACC/ p.T1093_T1095del | 4.51×10^-5^ | 4.87×10^-4^ | PS3+PM4 | LP | - | - | - | - | - | - | - | - | - | - |
| c.3356C>T/p.T1119I | 3.91×10^-4^ | 5.39×10^-3^ | PS3+PP2+PP3 | LP | D  (0.0) | D  (0.000) | D  (1.000) | D  (0.988) | D  (0.999) | D  (0.042) | D  (23.2) | D  (1.000) | C  (5.08) | C  (8.967) |
| c.3757C>T/p.R1253C | 1.50×10^-4^ | 1.74×10^-3^ | PS3+PP2+PP3 | LP | T  (0.206) | N  (0.204) | P  (1) | T  (0.069) | T  (0.813) | T  (0.005) | T  (0.381) | D  (1.000) | NC  (-6.23) | NC  (-3.366) |
| c.3790G>A/ p.G1264S | - | - | PS3+PM2+PP2+PP3 | LP | T  (0.902) | N  (0.001) | P  (1) | T  (0.032) | T  (0.314) | D  (0.026) | T  (0.001) | D  (1.000) | NC  (-1.68) | NC  (-0.059) |
| c.3845C>T/ p.S1282L | 3.88×10^-5^ | 3.69×10^-4^ | PS3+PP2+PP3 | LP | D  (0.003) | N  (0.089) | P  (1) | T  (0.289) | D  (0.997) | D  (0.090) | T  (11.36) | D  (1.000) | C  (4.54) | C  (2.248) |
| c.4217C>T/ p.A1406V | 2.23×10^-5^ | 0 | PS3+PP2+PP3 | LP | T  (0.543) | N  (0.017) | P  (1) | D  (0.686) | T  (0.938) | T  (0.009) | T  (3.367) | D  (1.000) | C  (3.77) | NC  (0.879) |
| c.4384G>A/ p.D1462N | - | - | PM2+PP2+PP3 | VUS | D  (0.003) | N  (0.006) | P  (0.998) | D  (0.951) | D  (0.998) | D  (0.040) | D  (31.0) | D  (1.000) | C  (5.94) | C  (3.970) |

ACMG, American College of Medical Genetics and Genomics guideline; MAF, minor allele frequency from Genome Aggregation Database (gnomAD); MAF-EAS, minor allele frequency from Genome Aggregation Database-East Asian population; LoF, Loss-of-function; C, conserved; D, damaging or deleterious or disease_causing; N, neutral; NC, non-conserved; P, Polymorphism; T, tolerable; VUS, Variants of uncertain significance.

PM2, absent from controls (or at extremely low frequency if recessive) in gnomAD; PM4 Protein length changes as a result of in-frame deletions/insertions in a nonrepeat region or stop-loss variants; PP2 Missense variant in a gene that has a low rate of benign missense variation and in which missense variants are a common mechanism of disease; PP3, multiple lines of computational evidence support a deleterious effect on the gene or gene product (conservation, evolutionary, splicing impact, etc.).

The damage of the *HCFC1* variants was predicted by 23 in silico algorithms (<http://varcards.biols.ac.cn/>). Owing to space limitations, only some typical results were indicated in this table.
