# Supplementary material for: HCFC1 variants in the proteolysis domain are associated with X‐linked idiopathic partial epilepsy: Exploring the underlying mechanism
Source: Clin Transl Med. 2023 Jun 1;13(6):e1289. doi: 10.1002/ctm2.1289 (PMC10235798; doi:10.1002/ctm2.1289)
Supplement: Supplementary file 6 — Supporting Information [file CTM2-13-e1289-s005.docx]

**Table S3. Clinical and genetic features of additional patients with *HCFC1* variants in the validation cohort.**

| Case | Sex | Mutation | Zygos-ity | Allele number in controls of gnomAD-all populations | |  | Allele number in controls of gnomAD- East Asian populations | | Origin | Domain | Age (yr) | Onset age | Clinical symptoms |
| --- | --- | --- | --- | --- | --- | --- | --- | --- | --- | --- | --- | --- | --- |
|  |  |  |  | **Hemi.** | **Homo.** |  | **Hemi.** | **Homo.** |  |  |  |  |  |
| A1 | M | c.1894A>G/p.I632V | Hemi. | - | - |  | - | - | Unknown | Basic | 4 | 8 mo | Focal seizures monthly and controlled by valproate, levetiracetam, and clonazepam; intellectual disability. EEG showed bilateral frontal sharps. |
| A2 | F | c.3356C>T/p.T1119I | Homo. | 11/86019 (0.00013) | 0/86019 |  | 11/7157 (0.0015) | 0/7157 | Unknown | Repeat 3 | 8.5 | 4 yr | Focal seizures |
| A3 | M | c.3356C>T/p.T1119I | Hemi. | 11/86019 (0.00013) | 0/86019 |  | 11/7157 (0.0015) | 0/7157 | Unknown | Repeat 3 | 3 | 2 yr | Seizures with only two attacks. |
| A4 | M | c.3356C>T/p.T1119I | Hemi. | 11/86019 (0.00013) | 0/86019 |  | 11/7157 (0.0015) | 0/7157 | Unknown | Repeat 3 | 3 | 1 yr | Febrile seizures for several times and controlled by levetiracetam. |
| A5 | M | c.3563C>T/p.S1188L | Hemi. | 1/39286 (0.00003) | 0/39286 |  | 0/3198 | 0/3198 | Maternal | HCF-1_PRO_ | 7 | 5 yr | Rolandic epilepsy, only three attacks during sleep, seizure free for one year under the treatment of valproate and lamotrigine. EEG showed bilateral Rolandic discharge. |
| A6 | M | c.3705T>G/p.H1235Q | Hemi. | - | - |  | - | - | Maternal | HCF-1_PRO_ | 18 | 9 yr | Focal seizures and tonic-clonic seizures that controlled by oxcarbazepine; intellectual disability. EEG showed multifocal discharge. |
| A7 | M | c.3734C>G/p.S1245C | Hemi. | 3/82871 (0.00004) | 0/82871 |  | 3/6960 (0.00043) | 0/6960 | Unknown | HCF-1_PRO_ | 4 | 2.5 yr | Focal seizures with several attacks, that controlled by valproate, levetiracetam, and lamotrigine. EEG showed epileptic discharge in bilateral anterior region. |
| A8 | F | c.3757C>T/p.R1253C | Homo. | 4/84220 (0.00005) | 0/84220 |  | 3/7145 (0.00042) | 0/7145 | Unknown | HCF-1_PRO_ | 4.5 | 2 yr | Three tonic-clonic seizures in half a year. Her father had a seizure attack in his childhood. |
| A9 | M | c.3757C>T/p.R1253C | Hemi. | 4/84220 (0.00005) | 0/84220 |  | 3/7145 (0.00042) | 0/7145 | Unknown | HCF-1_PRO_ | 7 | 6 mo | Frequent daily focal seizures, intellectual disability, congenital heart disease |
| A10 | M | c.3757C>T/p.R1253C | Hemi. | 4/84220 (0.00005) | 0/84220 |  | 3/7145 (0.00042) | 0/7145 | Maternal | HCF-1_PRO_ | 4 | First day of age | Focal seizures at a frequency of 6-10 times/day; intellectual disability. EEG showed multifocal discharge. The brain MRI showed bilateral ventricles enlarged and absence of corpus callosum. |
| A11 | M | c.3995C>T/p.T1332M | Hemi. | - | - |  | - | - | Unknown | Repeat 5 | 6 | 9 mo | Seizures controlled by levetiracetam and clonazepam, speech lag. |
| A12 | M | c.4135G>A/p.D1379N | Hemi. | 1/76265 (0.00001) | 0/76265 |  | 1/6589 (0.00015) | 0/6589 | Unknown | HCF-1_PRO_ | 2 | 10 mo | Complex partial seizures occasionally. |
| A13 | M | c.4135G>A/p.D1379N | Hemi. | 1/76265 (0.00001) | 0/76265 |  | 1/6589 (0.00015) | 0/6589 | Maternal | HCF-1_PRO_ | 9.5 | 6.5 yr | Seizures monthly and controlled by levetiracetam. EEG showed bilateral Rolandic discharge |

F, female; Hemi., hemizygous; Homo., homozygous; M, male; mo, months; yr, years.
