# Supplementary material for: HCFC1 variants in the proteolysis domain are associated with X‐linked idiopathic partial epilepsy: Exploring the underlying mechanism
Source: Clin Transl Med. 2023 Jun 1;13(6):e1289. doi: 10.1002/ctm2.1289 (PMC10235798; doi:10.1002/ctm2.1289)
Supplement: Supplementary file 7 — Supporting Information [file CTM2-13-e1289-s002.docx]

**Table S4. Genetic and phenotypic information of patients with *HCFC1* variants.**

| **Variants^a^** | **Case (AFM)^b^** | **Sex** | **Inheritance** | **Location** | **Metabolism** | **ID** | **DD** | **ASD** | **Dysmorphic** | **Seizures** | **Ep prognosis** | **Reference** |
| --- | --- | --- | --- | --- | --- | --- | --- | --- | --- | --- | --- | --- |
| c.-970T>C/- | 1 (12) | M | Maternal | 5'UTR | - | + | + | + | + | + (1/12) | Improved | Huang, 2012 |
| c.202C>G/p.Q68E | 1  1 | M  M | Unknown  Maternal | K1  K1 | +  + | +  NA | +  NA | -  NA | +  NA | +  + | Intractable  Early death (1) | Yu, 2013  Shen, 2021 |
| c.217G>A/p.A73T | 1 | M | Unknown | K1 | + | + | + | - | - | + | Intractable | Yu, 2013 |
| c.218C>T/p.A73V | 1 (2)  2 | M | Maternal  De novo (1)  Unknown (1) | K1 | + | + | + | + | + | + (3/4) | Intractable or  early death (1) | Yu, 2013; Redin, 2014 |
| c.307T>C/p.Y103H | 1 (2) | M | Maternal | K2 | + | + | + | - | + | + (1/2) | Intractable and death (1), miscarried (1) | Gerard, 2015 |
| c.343G>A/p.A115T | 1 | M | Unknown | K2 | + | + | + | - | + | +? | Early death (1) | Yu, 2013 |
| c.344C>T/p.A115V | 10 | M | Maternal (2)  Unknown (8) | K2 | + | + | + | - | + | + (10/10) | Intractable | Yu, 2013; Scalais, 2017 |
| c.674G>A/p.S225N | 1 (4) | M | Maternal | K4 | - | + | + | - | - | - | - | Huang, 2012 |
| c.1024G>A/p.G342S | 1 | M | Segregated | Fn3 | NA | + | NA | NA | NA | NA | NA | Niranjan, 2015 |
| c.1429G>A/p.A477T | 2 | M  ? | Maternal | Basic | - | + (2/2) | + | - | + (1/2) | + (2/2) | Seizure free;  NA | Jolly, 2015; Bowling, 2017 |
| c.1781_1803+3del26insCA | 1 (5) | M (3)  F (2) | Maternal | Basic | - | + (5/5) | + (4/5) | - | + (3/5) | + (2/5) | Controlled | Wongkittichote, 2021 |
| c.1894A>G/p.I632V | 1 | M | Unknown | Basic | - | + | - | - | - | + | Seizure free | This study |
| c.2324G>A/p.S775L | 1 | M | Maternal | Basic | NA | + | NA | NA | NA | NA | NA | Tarpey, 2009 |
| c.2492C>T/p.T831I | 1 | M | Maternal | Basic | - | + | + | - | + | - | - | Fitzgerald, 2015 |
| c.2564G>T/ p.R855L | 1 | M | Unknown | Basic | NA | + | NA | NA | NA | NA | NA | Stranneheim,2021 |
| c.2590G>A/p.A864T | 1 | M | Maternal | Basic | NA | + | NA | NA | NA | NA | NA | Tarpey, 2009 |
| c.2626G>A/p.G876S | 2 | M | Maternal | Basic | - | + (1/2) | + (1/2) | + (1/2) | + (1/2) | + (1/2) | Seizure free | Piton, 2011; Huang, 2012 |
| c.2690G>A/p.A897V | 1 (2) | M | Maternal | Basic | - | +(2/2) | +(2/2) | - | + (2/2) | - | - | Koufaris, 2016 |
| c.2860G>A/p.V954M | 1 | M | Maternal | Basic | - | + | NA | + | - | + | Intractable | Long, 2019 |
| **c.3277_3285delACCGCCACC/ p.T1093_T1095del** | **1** | **M** | **Maternal** | **Repeat 2** | **-** | **-** | **-** | **-** | **-** | **+** | **Seizure free** | **This study** |
| **c.3356C>T/p.T1119I** | **8** | **M (7)**  **F (1)** | **Maternal (5)**  **Unknown (3)** | **Repeat 3** | **-** | **+(2/8)** | **-** | **-** | **-** | **+ (8/8)** | **Seizure free or controlled** | **This study** |
| **c.3563C>T/p.S1188L** | **1** | **M** | **Maternal** | **HCF-1_PRO_** | **-** | **-** | **-** | **-** | **-** | **+** | **Seizure free** | **This study** |
| c. 3596G>C/p.R1199P | 1 | M | XR | HCF-1_PRO_ | - | + | - | + | + | - | - | Wang, 2018 |
| **c.3705T>G/p.H1235Q** | **1** | **M** | **Maternal** | **HCF-1_PRO_** | **-** | **+** | **-** | **-** | **-** | **+** | **Seizure free** | **This study** |
| **c.3734C>G/p.S1245C** | **1** | **M** | **Unknown** | **HCF-1_PRO_** | **-** | **-** | **-** | **-** | **-** | **+** | **Seizure free** | **This study** |
| **c.3757C>T/p.R1253C** | **4** | **M (3)**  **F (1)** | **Maternal (2)**  **Unknown (2)** | **HCF-1_PRO_** | **-** | **+(3/4)** | **-** | **-** | **-** | **+ (4/4)** | **Controlled** | **This study** |
| **c.3790G>A/p.G1264S** | **1** | **M** | **Maternal** | **HCF-1_PRO_** | **-** | **-** | **-** | **-** | **-** | **+** | **Seizure free** | **This study** |
| **c.3845C>T/ p.S1282L** | **2** | **M** | **Maternal** | **HCF-1_PRO_** | **-** | **+(1/2)** | **-** | **-** | **-** | **+ (2/2)** | **Seizure free** | **This study Guo, 2021** |
| **c.3995C>T/p.T1332M** | **1** | **M** | **Unknown** | **Repeat 5** | **-** | **-** | **-** | **+** | **-** | **+** | **Seizure free** | **This study** |
| **c.4135G>A/p.D1379N** | **2** | **M** | **Maternal (1)**  **Unknown (1)** | **HCF-1_PRO_** | **-** | **-** | **-** | **-** | **-** | **+ (2/2)** | **Controlled** | **This study** |
| **c.4217C>T/ p.A1406V** | **1** | **M** | **Maternal** | **HCF-1_PRO_** | **-** | **-** | **-** | **-** | **-** | **+** | **Seizure free** | **This study** |
| **c.4315A>C/** **p.N1439H** | **1** | **M** | **Maternal** | **Acidic** | **-** | **+** | **NA** | **NA** | **NA** | **-** | **-** | **Klee, 2021** |
| **c.4384G>A/ p.D1462N** | **1** | **M** | **Maternal** | **Acidic** | **-** | **+** | **-** | **-** | **-** | **+** | **Seizure free** | **This study** |
| c.4442C>T/p.T1481M | 2 | M | **Maternal** Unknown | Acidic | - | +(2/2) | +(1/2) | - | - | + (1/2) | NA | Abdrabo, 2020  Guo, 2021 |
| c.5048C>G/p.P1683R | 1 | M | De novo | Acidic | - | + | + | + | + | + | NA | Bowling, 2017 |
| c.5267C>T/p.A1756V | 1 | M | Maternal | Acidic | - | + | + | - | - | - | - | Huang, 2012 |
| c.5305G>A/ p.V1769M | 1 | M | Maternal | Acidic | - | + | NA | + | NA | NA | NA | Xiong, 2019 |
| c.5379+2T>C/- | 1 | M | Maternal | - | NA | + | NA | NA | NA | NA | NA | Tarpey, 2009 |
| c.5764G>A/p.E1922K | 1 | M | De novo | Fn3 | - | + | NA | NA | NA | NA | NA | Martin, 2021 |
| c.5843T>C/ p.F1948S | 1 | M | De novo | Fn3 | - | + | NA | NA | NA | NA | NA | Martin, 2021 |
| c.5860G>A/p.G1954R | 1 | M | De novo | Fn3 | NA | + | NA | NA | NA | NA | NA | Farwell, 2015 |
| c.5938A>G/ p.I1980V | 1 | M | De novo | Fn3 | - | + | NA | NA | NA | NA | NA | Martin, 2021 |
| c.6046C>T/p.R2016W | 1 (2) | M | Maternal | NLS | - | +(2/2) | +(2/2) | - | + (2/2) | + (1/2) | Seizure free | Jolly, 2015 |
| Variants flagged as benign or with uncertain significance | | | | | | | | | | | | |
| c.722C>T/p.T241M | 1 (2) | F | Maternal | K4 | NA | NA | NA | NA | NA | NA | NA | Piton, 2011 |
| c.2563C>T/p.R855C | 1 | ? | De novo | Basic | NA | NA | NA | NA | NA | NA | NA | Kosmicki, 2017 |
| c.3794C>T/p.S1265L | 1 | F | Unknown | HCF-1_PRO_ | - | + | + | - | + | - | - | Sobreira, 2017 |
| c.4475C>T/p.P1492L | 1 | F | Unknown | Acidic | + | - | + | - | - | + | NA | Abdrabo, 2020 |
| c.4475C>G/p.P1492R | 1 | M | Unknown | Acidic | + | NA | NA | NA | NA | NA | Neonatal death | Zhang, 2019 |

To explore the genotype-phenotype association, all *HCFC1* mutations and related phenotypes were retrieved from the PubMed and HGMD database (http://www.hgmd.cf.ac.uk/ac/index.php; version: HGMD Professional 2022.3) till Oct 2022. All *HCFC1* mutations were annotated based on the transcript NM_005334.2.

ASD, autism spectrum disorder; DD, developmental delay; Ep, epilepsy; F, female; AFM, affected family member; HCF-1_PRO_, proteolysis domain; ID, intellectual disability; K, kelch domain; M, male; NA, not available; NLS, nuclear localization signal.

+?, the patient showed abnormal EEG.

**^a^** The mutations were annotated based on the transcript NM_005334.2, c.-970T>C was previously annotated as chrX: 152890455A>G, S775L as S678L, A864T as A767T, G876S as G779S, c.5379+2 C>T as c.5088+2 C>T, T241M as T142M.

^b^ Affected family members were indicated by numbers in parentheses.

R855C was related to congenital heart disease.

T241M was reported in two sisters with SCZ.

Heterozygous S1265L mutation was reported in one female individual with Kabuki syndrome, which is characterized by intellectual disability, dysmorphic face, growth retardation, and immune dysfunction.

**Supplementary references**

Abdrabo LS, Watkins D, Wang SR, Lafond-Lapalme J, Riviere JB, Rosenblatt DS. (2020) Genome and RNA sequencing in patients with methylmalonic aciduria of unknown cause. *Genet Med* 22:432-436.

Bowling KM, Thompson ML, Amaral MD, Finnila CR, Hiatt SM, Engel KL, Cochran JN, Brothers KB, East KM, Gray DE, Kelley WV, Lamb NE, Lose EJ, Rich CA, Simmons S, Whittle JS, Weaver BT, Nesmith AS, Myers RM, Barsh GS, Bebin EM, Cooper GM. (2017) Genomic diagnosis for children with intellectual disability and/or developmental delay. *Genome Med* 9:43.

Farwell KD, Shahmirzadi L, El-Khechen D, Powis Z, Chao EC, Tippin Davis B, Baxter RM, Zeng W, Mroske C, Parra MC, Gandomi SK, Lu I, Li X, Lu H, Lu HM, Salvador D, Ruble D, Lao M, Fischbach S, Wen J, Lee S, Elliott A, Dunlop CL, Tang S. (2015) Enhanced utility of family-centered diagnostic exome sequencing with inheritance model-based analysis: results from 500 unselected families with undiagnosed genetic conditions. *Genet Med* 17:578-586.

Fitzgerald TW, Gerety SS, Jones WD, Kogelenberg Mv, King DA. (2015) Large-scale discovery of novel genetic causes of developmental disorders. *Nature* 519:223-228.

Gerard M, Morin G, Bourillon A, Colson C, Mathieu S, Rabier D, Billette de Villemeur T, Ogier de Baulny H, Benoist JF. (2015) Multiple congenital anomalies in two boys with mutation in HCFC1 and cobalamin disorder. *Eur J Med Genet* 58:148-153.

Guo YX, Ma HX, Zhang YX, Chen ZH, Zhai QX. (2021) Whole-Exome Sequencing for Identifying Genetic Causes of Intellectual Developmental Disorders. *Int J Gen Med* 14:1275-1282.

Huang L, Jolly LA, Willis-Owen S, Gardner A, Kumar R, Douglas E, Shoubridge C, Wieczorek D, Tzschach A, Cohen M, Hackett A, Field M, Froyen G, Hu H, Haas SA, Ropers HH, Kalscheuer VM, Corbett MA, Gecz J. (2012) A noncoding, regulatory mutation implicates HCFC1 in nonsyndromic intellectual disability. *Am J Hum Genet* 91:694-702.

Jolly LA, Nguyen LS, Domingo D, Sun Y, Barry S, Hancarova M, Plevova P, Vlckova M, Havlovicova M, Kalscheuer VM, Graziano C, Pippucci T, Bonora E, Sedlacek Z, Gecz J. (2015) HCFC1 loss-of-function mutations disrupt neuronal and neural progenitor cells of the developing brain. *Hum Mol Genet* 24:3335-3347.

Klee EW, Cousin MA, Pinto EVF, Morales-Rosado JA, Macke EL, Jenkinson WG, Ferrer A, Schultz-Rogers LE, Olson RJ, Oliver GR, Sigafoos AN, Schwab TL, Zimmermann MT, Urrutia RA, Kaiwar C, Gupta A, Blackburn PR, Boczek NJ, Prochnow CA, Lowy RJ, Mulvihill LA, McAllister TM, Aoudia SL, Kruisselbrink TM, Gunderson LB, Kemppainen JL, Fisher LJ, Tarnowski JM, Hager MM, Kroc SA, Bertsch NL, Agre KE, Jackson JL, Macklin-Mantia SK, Murphree MI, Rust LM, Summer Bolster JM, Beck SA, Atwal PS, Ellingson MS, Barnett SS, Rasmussen KJ, Lahner CA, Niu Z, Hasadsri L, Ferber MJ, Marcou CA, Clark KJ, Pichurin PN, Deyle DR, Morava-Kozicz E, Gavrilova RH, Dhamija R, Wierenga KJ, Lanpher BC, Babovic-Vuksanovic D, Farrugia G, Schimmenti LA, Stewart AK, Lazaridis KN. (2021) Impact of integrated translational research on clinical exome sequencing. *Genet Med* 23:498-507.

Kosmicki JA, Samocha KE, Howrigan DP, Sanders SJ, Slowikowski K, Lek M, Karczewski KJ, Cutler DJ, Devlin B, Roeder K, Buxbaum JD, Neale BM, MacArthur DG, Wall DP, Robinson EB, Daly MJ. (2017) Refining the role of de novo protein-truncating variants in neurodevelopmental disorders by using population reference samples. *Nat Genet* 49:504-510.

Koufaris C, Alexandrou A, Tanteles GA, Anastasiadou V, Sismani C. (2016) A novel HCFC1 variant in male siblings with intellectual disability and microcephaly in the absence of cobalamin disorder. *Biomed Rep* 4:215-218.

Long S, Zhou H, Li S, Wang T, Ma Y, Li C, Zhou Y, Zhou S, Wu B, Wang Y. (2019) The Clinical and Genetic Features of Co-occurring Epilepsy and Autism Spectrum Disorder in Chinese Children. *Front Neurol* 10:505.

Martin HC, Gardner EJ, Samocha KE, Kaplanis J, Akawi N, Sifrim A, Eberhardt RY, Tavares ALT, Neville MDC, Niemi MEK, Gallone G, McRae J, Deciphering Developmental Disorders S, Wright CF, FitzPatrick DR, Firth HV, Hurles ME. (2021) The contribution of X-linked coding variation to severe developmental disorders. *Nat Commun* 12:627.

Niranjan TS, Skinner C, May M, Turner T, Rose R, Stevenson R, Schwartz CE, Wang T. (2015) Affected kindred analysis of human X chromosome exomes to identify novel X-linked intellectual disability genes. *PLoS One* 10:e0116454.

Piton A, Gauthier J, Hamdan FF, Lafreniere RG, Yang Y, Henrion E, Laurent S, Noreau A, Thibodeau P, Karemera L, Spiegelman D, Kuku F, Duguay J, Destroismaisons L, Jolivet P, Cote M, Lachapelle K, Diallo O, Raymond A, Marineau C, Champagne N, Xiong L, Gaspar C, Riviere JB, Tarabeux J, Cossette P, Krebs MO, Rapoport JL, Addington A, Delisi LE, Mottron L, Joober R, Fombonne E, Drapeau P, Rouleau GA. (2011) Systematic resequencing of X-chromosome synaptic genes in autism spectrum disorder and schizophrenia. *Mol Psychiatry* 16:867-880.

Redin C, Gerard B, Lauer J, Herenger Y, Muller J, Quartier A, Masurel-Paulet A, Willems M, Lesca G, El-Chehadeh S, Le Gras S, Vicaire S, Philipps M, Dumas M, Geoffroy V, Feger C, Haumesser N, Alembik Y, Barth M, Bonneau D, Colin E, Dollfus H, Doray B, Delrue MA, Drouin-Garraud V, Flori E, Fradin M, Francannet C, Goldenberg A, Lumbroso S, Mathieu-Dramard M, Martin-Coignard D, Lacombe D, Morin G, Polge A, Sukno S, Thauvin-Robinet C, Thevenon J, Doco-Fenzy M, Genevieve D, Sarda P, Edery P, Isidor B, Jost B, Olivier-Faivre L, Mandel JL, Piton A. (2014) Efficient strategy for the molecular diagnosis of intellectual disability using targeted high-throughput sequencing. *J Med Genet* 51:724-736.

Scalais E, Osterheld E, Weitzel C, De Meirleir L, Mataigne F, Martens G, Shaikh TH, Coughlin CR, 2nd, Yu HC, Swanson M, Friederich MW, Scharer G, Helbling D, Wendt-Andrae J, Van Hove JLK. (2017) X-Linked Cobalamin Disorder (HCFC1) Mimicking Nonketotic Hyperglycinemia With Increased Both Cerebrospinal Fluid Glycine and Methylmalonic Acid. *Pediatr Neurol* 71:65-69.

Shen Y, Hu Z, Yang J, Yang R, Huang X. (2021) A case of methylmalonic acidemia and homocysteinemia cblX type with negative tandem mass spectrometry testing. *Zhejiang Da Xue Xue Bao Yi Xue Ban* 50:795-798.

Sobreira N, Brucato M, Zhang L, Ladd-Acosta C, Ongaco C, Romm J, Doheny KF, Mingroni-Netto RC, Bertola D, Kim CA, Perez AB, Melaragno MI, Valle D, Meloni VA, Bjornsson HT. (2017) Patients with a Kabuki syndrome phenotype demonstrate DNA methylation abnormalities. *Eur J Hum Genet* 25:1335-1344.

Stranneheim H, Lagerstedt-Robinson K, Magnusson M, Kvarnung M, Nilsson D, Lesko N, Engvall M, Anderlid BM, Arnell H, Johansson CB, Barbaro M, Bjorck E, Bruhn H, Eisfeldt J, Freyer C, Grigelioniene G, Gustavsson P, Hammarsjo A, Hellstrom-Pigg M, Iwarsson E, Jemt A, Laaksonen M, Enoksson SL, Malmgren H, Naess K, Nordenskjold M, Oscarson M, Pettersson M, Rasi C, Rosenbaum A, Sahlin E, Sardh E, Stodberg T, Tesi B, Tham E, Thonberg H, Tohonen V, von Dobeln U, Vassiliou D, Vonlanthen S, Wikstrom AC, Wincent J, Winqvist O, Wredenberg A, Ygberg S, Zetterstrom RH, Marits P, Soller MJ, Nordgren A, Wirta V, Lindstrand A, Wedell A. (2021) Integration of whole genome sequencing into a healthcare setting: high diagnostic rates across multiple clinical entities in 3219 rare disease patients. *Genome Med* 13:40.

Tarpey PS, Smith R, Pleasance E, Whibley A, Edkins S, Hardy C, O'Meara S, Latimer C, Dicks E, Menzies A, Stephens P, Blow M, Greenman C, Xue Y, Tyler-Smith C, Thompson D, Gray K, Andrews J, Barthorpe S, Buck G, Cole J, Dunmore R, Jones D, Maddison M, Mironenko T, Turner R, Turrell K, Varian J, West S, Widaa S, Wray P, Teague J, Butler A, Jenkinson A, Jia M, Richardson D, Shepherd R, Wooster R, Tejada MI, Martinez F, Carvill G, Goliath R, de Brouwer AP, van Bokhoven H, Van Esch H, Chelly J, Raynaud M, Ropers HH, Abidi FE, Srivastava AK, Cox J, Luo Y, Mallya U, Moon J, Parnau J, Mohammed S, Tolmie JL, Shoubridge C, Corbett M, Gardner A, Haan E, Rujirabanjerd S, Shaw M, Vandeleur L, Fullston T, Easton DF, Boyle J, Partington M, Hackett A, Field M, Skinner C, Stevenson RE, Bobrow M, Turner G, Schwartz CE, Gecz J, Raymond FL, Futreal PA, Stratton MR. (2009) A systematic, large-scale resequencing screen of X-chromosome coding exons in mental retardation. *Nat Genet* 41:535-543.

Wang X, Shen X, Fang F, Ding CH, Zhang H, Cao ZH, An DY. (2018) Phenotype-Driven Virtual Panel Is an Effective Method to Analyze WES Data of Neurological Disease. *Front Pharmacol* 9:1529.

Wongkittichote P, Wegner DJ, Shinawi MS. (2021) Novel exon-skipping variant disrupting the basic domain of HCFC1 causes intellectual disability without metabolic abnormalities in both male and female patients. *J Hum Genet* 66:717-724.

Xiong J, Chen S, Pang N, Deng X, Yang L, He F, Wu L, Chen C, Yin F, Peng J. (2019) Neurological Diseases With Autism Spectrum Disorder: Role of ASD Risk Genes. *Front Neurosci* 13:349.

Yu HC, Sloan JL, Scharer G, Brebner A, Quintana AM, Achilly NP, Manoli I, Coughlin CR, 2nd, Geiger EA, Schneck U, Watkins D, Suormala T, Van Hove JL, Fowler B, Baumgartner MR, Rosenblatt DS, Venditti CP, Shaikh TH. (2013) An X-linked cobalamin disorder caused by mutations in transcriptional coregulator HCFC1. *Am J Hum Genet* 93:506-514.

Zhang W, Yang Y, Peng W, Chang J, Mei Y, Yan L, Chen Y, Wei X, Liu Y, Wang Y, Feng Z. (2019) A 7-Year Report of Spectrum of Inborn Errors of Metabolism on Full-Term and Premature Infants in a Chinese Neonatal Intensive Care Unit. *Front Genet* 10:1302.
